# Supplementary material for: Dysregulated transforming growth factor-beta mediates early bone marrow dysfunction in diabetes
Source: Commun Biol. 2022 Oct 28;5:1145. doi: 10.1038/s42003-022-04112-2 (PMC9616825; doi:10.1038/s42003-022-04112-2)
Supplement: Supplementary file 2 — Supplementary Information [file 42003_2022_4112_MOESM2_ESM.pdf]

Dysregulated transforming growth factor-beta mediates early bone marrow dysfunction in diabetes

Jina J. Y. Kum[1], Christopher J. Howlett[1,2,3], and Zia A. Khan[1,3,4]

[1] Pathology and Laboratory Medicine, Schulich School of Medicine & Dentistry, Western University, London, Ontario, Canada
[2] Pathology and Laboratory Medicine, London Health Sciences Centre, London, Ontario, Canada
[3] Lawson Research Institute, London, Ontario, Canada
[4] Division of Genetics & Development, Children's Health Research Institute, London, Ontario Canada.

Short title: TGFB in bone marrow dysfunction in diabetes

Supplementary File:
Supplementary Figures and Tables: 22 figures and 11 tables.

Supplementary Table S1: Media formulation for insulin-independent lipid accumulation in cells.

Component				Media				
	**Catalogue #, Vendor**	**Diluent**	**[Stock: Final]**	**Control media**	**idADP**	**idADP + HG**	**idADP + LIN/OL**	**idADP + LIN/OL + HG**
DMEM, low glucose, pyruvate	11885084, Thermo Fisher	-	-	+	+	+	+	+
Fetal Bovine Serum, qualified, heat inactivated	12484028, Thermo Fisher	-	100%: 10%	+	+	+	+	+
Dexamethasone -water soluble	D2915, Sigma-Aldrich	EtOH	1 mM: 1 μM	-	+	+	+	+
Indomethacin	I7378, Sigma-Aldrich	EtOH	50 mM: 50 μM	-	+	+	+	+
3-Isobutyl-1-methylxanthine	I7018, Sigma-Aldrich	EtOH	50 mM: 0.5 mM	-	+	+	+	+
Dextrose, anhydrous	CABDH0230, VWR chemicals	DMEM, low glucose	1 M: 20 mM	-	-	+	-	+
Linoleic Acid-Oleic Acid-Albumin	L9655, Sigma-Aldrich	-	100X: 2X	-	-	-	+	+
Antibiotic Antimycotic	30-004-CI, Corning	-	100X: 1X	+	+	+	+	+

Abbreviations: ADP = adipogenesis induction media; idADP = insulin-deficient adipogenesis induction media; EtOH = ethanol; HG = high glucose (25 mmol/L); LIN/OL = linoleic acid-oleic acid.

Supplementary Table S2: Gene ontology (GO) biological processes from target genes that are upregulated in bm-MPCs subjected to 1) adipogenic differentiation, and 2) adipogenic differentiation media with TGFB1 and TAK1 inhibitor.

Gene ontology (GO) biological processes enriched

Biological Process Term		p-value
GO:0055114	oxidation-reduction process	1.27E-05
GO:0006635	fatty acid beta-oxidation	8.83E-05
GO:0006098	pentose-phosphate shunt	1.70E-04
GO:0015909	long-chain fatty acid transport	2.25E-04
GO:0006629	lipid metabolic process	2.34E-04
GO:0042493	response to drug	0.001295
GO:0006631	fatty acid metabolic process	0.002047
GO:0010886	positive regulation of cholesterol storage	0.002167
GO:0043524	negative regulation of neuron apoptotic process	0.002535
GO:0034383	low-density lipoprotein particle clearance	0.002869
GO:0055085	transmembrane transport	0.003987
GO:0098869	cellular oxidant detoxification	0.006003
GO:0042953	lipoprotein transport	0.010259
GO:0006646	phosphatidylethanolamine biosynthetic process	0.010259
GO:0050731	positive regulation of peptidyl-tyrosine phosphorylation	0.010409
GO:0010595	positive regulation of endothelial cell migration	0.011979
GO:0070542	response to fatty acid	0.013109
GO:0051968	positive regulation of synaptic transmission, glutamatergic	0.016261
GO:0022904	respiratory electron transport chain	0.017946
GO:0035902	response to immobilization stress	0.019702
GO:0007173	epidermal growth factor receptor signaling pathway	0.020296
GO:0009051	pentose-phosphate shunt, oxidative branch	0.020617
GO:0042412	taurine biosynthetic process	0.020617
GO:0019322	pentose biosynthetic process	0.020617
GO:0019915	lipid storage	0.025376
GO:0033344	cholesterol efflux	0.027397
GO:0006656	phosphatidylcholine biosynthetic process	0.027397
GO:0032496	response to lipopolysaccharide	0.028214
GO:0006549	isoleucine metabolic process	0.030767
GO:0000255	allantoin metabolic process	0.030767
GO:0032868	response to insulin	0.032316
GO:0051881	regulation of mitochondrial membrane potential	0.033832
GO:0070374	positive regulation of ERK1 and ERK2 cascade	0.035667
GO:0006642	triglyceride mobilization	0.040812
GO:0046449	creatinine metabolic process	0.040812

GO:0006573	valine metabolic process	0.040812
GO:0032869	cellular response to insulin stimulus	0.045823
GO:0019221	cytokine-mediated signaling pathway	0.047437

Supplementary Table S3: Gene ontology (GO) biological processes from target genes that are downregulated in bm-MPCs subjected to 1) adipogenic differentiation, 2) adipogenic differentiation media with TGFB1 and TAK1 inhibitor.

**Biological Process Term**		**p-value**
GO:0007017	microtubule-based process	2.04E-04
GO:0098609	cell-cell adhesion	3.86E-04
GO:0045892	negative regulation of transcription, DNA-templated	0.002616
GO:0090190	positive regulation of branching involved in ureteric bud morphogenesis	0.010318
GO:0001649	osteoblast differentiation	0.010401
GO:0032922	circadian regulation of gene expression	0.011277
GO:1900275	negative regulation of phospholipase C activity	0.016251
GO:0042384	cilium assembly	0.018749
GO:0008652	cellular amino acid biosynthetic process	0.018895
GO:0007265	Ras protein signal transduction	0.019547
GO:0060271	cilium morphogenesis	0.025299
GO:0045893	positive regulation of transcription, DNA-templated	0.025357
GO:0001501	skeletal system development	0.025888
GO:0007155	cell adhesion	0.034578
GO:0030177	positive regulation of Wnt signaling pathway	0.034745
GO:0060071	Wnt signaling pathway, planar cell polarity pathway	0.039448
GO:0055129	L-proline biosynthetic process	0.040138
GO:0007411	axon guidance	0.041325
GO:0006561	proline biosynthetic process	0.047971

Supplementary Table S4: Gene sets enriched in bm-MPCs exposed to control and adipogenic differentiation media (ADP).

Condition	Gene Set	Size	NES	p-value	FDR
ADP	Adipogenesis	187	2.658365	0	0
	Fatty Acid Metabolism	152	2.406275	0	0
	Xenobiotic Metabolism	191	1.972802	0	0
	Cholesterol Homeostasis	69	1.875002	0.002268	0.001414
	Oxidative Phosphorylation	176	1.788628	0	0.002573
	Peroxisome	99	1.587389	0.002375	0.014536
	Myc Targets V2	51	1.538435	0.026846	0.019612
	Bile Acid Metabolism	110	1.517775	0.00907	0.021075
	Androgen Response	92	1.491636	0.011494	0.024066
	Apical Surface	42	1.352561	0.090551	0.069815
	Reactive Oxygen Species Pathway	45	1.237397	0.147752	0.155475
	Pancreas Beta Cells	39	1.207659	0.209607	0.177652
	Angiogenesis	34	1.15252	0.263393	0.234508
	KRAS Signaling Dn	190	1.075596	0.266366	0.355486
	UV Response Up	154	1.057003	0.310945	0.369972
	Myc Targets V1	174	1.040503	0.340376	0.379482
	Spermatogenesis	129	1.016069	0.412301	0.409409
Control	Interferon Alpha Response	91	-2.11756	0	0
	Interferon Gamma Response	192	-2.11069	0	0
	Mitotic Spindle	193	-2.02258	0	2.75E-04
	Epithelial Mesenchymal Transition	192	-2.05704	0	3.67E-04
	TNFa Signaling via NFKB	192	-1.91684	0	6.50E-04
	Apical Junction	187	-1.86246	0	9.58E-04
	G2/M Checkpoint	180	-1.89573	0	0.001117
	Inflammatory Response	195	-1.77894	0	0.002031
	p53 Pathway	185	-1.76275	0	0.002376
	IL2 STAT5 Signaling	190	-1.72817	0	0.003334
	Protein Secretion	92	-1.68879	0.001825	0.004221
	Apoptosis	154	-1.69173	0	0.004408
	Unfolded Protein Response	97	-1.59861	0.003584	0.008945
	Coagulation	134	-1.59909	0	0.009584
	Estrogen Response Early	188	-1.60237	0.001689	0.010118
	E2F Targets	181	-1.52685	0.003565	0.016091
	UV Response Dn	132	-1.45121	0.017794	0.032148
	Complement	192	-1.43812	0.005474	0.034237
	KRAS Signaling Up	192	-1.42564	0.007156	0.036671
	Allograft Rejection	188	-1.39622	0.028319	0.045073
	Notch Signaling	31	-1.3424	0.102703	0.06793

	Hypoxia	192	-1.33269	0.021311	0.069599
	Estrogen Response Late	190	-1.24963	0.084577	0.132398
	TGF Beta Signaling	53	-1.21881	0.164602	0.160584
	PI3K AKt mTOR Signaling	100	-1.13364	0.207294	0.270295
	IL6 JAK STAT3 Signaling	85	-1.1351	0.242321	0.277975
	Heme Metabolism	185	-1.11822	0.216028	0.286274
	mTORC1 Signaling	192	-1.07734	0.289116	0.353933
	Myogenesis	190	-1.05333	0.328026	0.391704
	Glycolysis	192	-1.02774	0.389545	0.435953
	Hedgehog Signaling	34	-0.93503	0.551595	0.641383
	Wnt Beta Catenin Signaling	41	-0.86948	0.642857	0.781315
	DNA Repair	138	-0.76945	0.939338	0.927302

Supplementary Table S5: Gene sets enriched in bm-MPCs exposed to adipogenic differentiation media (ADP) with or without TGFB1.

Condition	Gene Set	Size	NES	p-value	FDR
ADP	Interferon Alpha Response	91	2.191829	0	0
	Adipogenesis	187	2.017069	0	0
	Xenobiotic Metabolism	191	1.895772	0	9.95E-04
	Fatty Acid Metabolism	152	1.871451	0	7.46E-04
	Bile Acid Metabolism	110	1.725169	0	0.002389
	Interferon Gamma Response	192	1.638922	0.003247	0.007206
	Apical Surface	42	1.406108	0.058076	0.061861
	Cholesterol Homeostasis	69	1.393647	0.039076	0.058884
	Complement	192	1.36985	0.016474	0.065428
	Peroxisome	99	1.352695	0.055077	0.069613
	Reactive Oxygen Species Pathway	45	1.271546	0.136937	0.128324
	KRAS Signaling Dn	190	1.237835	0.074919	0.152356
	IL6 JAK STAT3 Signaling	85	1.105878	0.272727	0.347811
	Myogenesis	190	1.088949	0.271829	0.357887
	Allograft Rejection	188	1.079157	0.288525	0.354051
	Coagulation	134	1.034265	0.390048	0.426172
	Oxidative phosphorylation	176	0.96039	0.56042	0.57492
	Spermatogenesis	129	0.90317	0.684474	0.679257
TGFB1	TGF Beta Signaling	53	-2.12261	0	0
	Unfolded Protein Response	97	-2.09296	0	0
	mTORC1 Signaling	192	-2.0696	0	0
	Epithelial Mesenchymal Transition	192	-2.03528	0	0
	Hypoxia	192	-2.01353	0	0
	Glycolysis	192	-1.94226	0	0
	TNFa Signaling via NFKB	192	-1.90582	0	6.19E-04
	G2/M Checkpoint	180	-1.77283	0	0.001843
	p53 Pathway	185	-1.62084	0	0.009627
	E2F Targets	181	-1.57311	0	0.015059
	IL2 STAT5 Signaling	190	-1.55387	0.002681	0.017656
	Hedgehog Signaling	34	-1.51943	0.033113	0.022994
	Inflammatory Response	195	-1.4207	0.010309	0.047972
	Estrogen Response Early	188	-1.35651	0.01061	0.078547
	Myc Targets V1	174	-1.33254	0.02267	0.088333
	UV Response Dn	132	-1.32667	0.031818	0.087379
	Wnt Beta Catenin Signaling	41	-1.30854	0.096552	0.097154
	Protein Secretion	92	-1.30152	0.06988	0.096993
	UV Response Up	154	-1.2737	0.062035	0.116662
	KRAS Signaling Up	192	-1.25186	0.046392	0.131479
	Androgen Response	92	-1.24269	0.088095	0.133734
	Apoptosis	154	-1.19927	0.099237	0.17918

	PI3K AKT mTOR Signaling	100	-1.18508	0.16152	0.189661
	Pancreas Beta Cells	39	-1.17789	0.210526	0.19274
	Mitotic Spindle	193	-1.17033	0.097297	0.194721
	Angiogenesis	34	-1.06676	0.339713	0.376011
	Apical Junction	187	-1.04731	0.315294	0.408796
	Estrogen Response Late	190	-1.01412	0.395161	0.475929
	Notch Signaling	31	-0.96412	0.486301	0.595359
	Myc Targets V2	51	-0.92005	0.586605	0.696053
	DNA Repair	138	-0.91215	0.698565	0.695802
	Heme Metabolism	185	-0.8993	0.716456	0.705125

Supplementary Table S6: Gene sets enriched in bm-MPCs exposed to adipogenic differentiation media and TGFB1 (TGFB1), with or without TAK1 inhibitor (TAK1i).

Condition	Gene Set	Size	NES	p-value	FDR
TAK1i	Adipogenesis	187	2.356003	0	0
	Fatty Acid Metabolism	152	2.139756	0	0
	Xenobiotic Metabolism	191	2.105543	0	0
	Interferon Alpha Response	91	2.003802	0	5.90E-04
	Cholesterol Homeostasis	69	1.897892	0	0.001146
	Complement	192	1.807903	0	0.001466
	p53 Pathway	185	1.811716	0	0.001711
	Reactive Oxygen Species Pathway	45	1.750302	0.001815	0.003332
	Oxidative Phosphorylation	176	1.693126	0	0.004221
	Bile Acid Metabolism	110	1.688197	0	0.004421
	Coagulation	134	1.576186	0.001712	0.012847
	Peroxisome	99	1.518858	0.00495	0.019752
	Interferon Gamma Response	192	1.491824	0.006462	0.023758
	Apoptosis	154	1.371015	0.025682	0.076556
	TNFa Signaling via NFKB	192	1.341559	0.027157	0.08899
	Apical Junction	187	1.347214	0.023256	0.090629
	Myogenesis	0.304618	0.030303	0.099075	0.79
	KRAS Signaling Up	0.298357	0.03882	0.110163	0.837
	UV Response Dn	0.312613	0.080268	0.121307	0.878
	Heme Metabolism	0.296372	0.0384	0.11931	0.883
	Estrogen Response Late	0.281801	0.094574	0.186286	0.98
	IL6 JAK STAT3 Signaling	0.316327	0.160584	0.211486	0.988
	Estrogen Response Early	0.270939	0.158516	0.259587	0.998
	Hypoxia	0.265547	0.178571	0.247971	0.999
	Apical Surface	0.344719	0.272381	0.257506	0.999
	Inflammatory Response	0.267227	0.189627	0.261144	0.999
	DNA Repair	0.272035	0.193493	0.267666	1
	UV Response Up	0.236674	0.470978	0.542542	1
	KRAS Signaling Dn	0.21025	0.728988	0.783954	1
	Androgen Response	0.228339	0.694946	0.809356	1
	Angiogenesis	0.258062	0.753271	0.901269	1
	Notch Signaling	0.252517	0.784133	0.942504	1
	Protein Secretion	0.146962	1	0.998405	1
TGFB1	G2/M Checkpoint	180	-1.95638	0	0
	Unfolded Protein Response	97	-1.86891	0	4.47E-04
	E2F Targets	181	-1.65828	0	0.009373
	Hedgehog Signaling	34	-1.58959	0.015119	0.014036
	Myc Targets V1	174	-1.48678	0.004878	0.027491

	Epithelial Mesenchymal Transition	192	-1.50045	0	0.028229
	Mitotic Spindle	193	-1.29085	0.028947	0.119671
	Myc Targets V2	51	-1.27503	0.124434	0.121352
	mTORC1 Signaling	192	-1.25921	0.040284	0.125785
	Spermatogenesis	129	-1.29553	0.061905	0.13208
	Pancreas Beta Cells	39	-1.18443	0.21322	0.206505
	Allograft Rejection	188	-1.16725	0.113208	0.215174
	IL2 STAT5 Signaling	190	-1.11181	0.176923	0.295312
	TGF Beta Signaling	53	-1.05677	0.367206	0.369251
	PI3K AKT mTOR Signaling	100	-1.05738	0.315673	0.393633
	Glycolysis	192	-1.0338	0.3625	0.395607
	Wnt Beta Catenin Signaling	41	-0.92121	0.594406	0.654772

Supplementary Table S7: qPCR primers for mouse genes.

Gene	Gene description	Chemistry	Source (Cat#, Reference)
Actb	Actin, beta	SYBR-green	Qiagen (QT00095242)
Actb	Actin, beta	Taqman	Thermo Fisher (Mm02619580_g1)
Adipoq	Adiponectin	SYBR-green	Thermo Fisher [1]
Angpt1	Angiopoietin 1	Taqman	Thermo Fisher (Mm00456503_m1)
Atp5f1	ATP synthase, H + transporting, mitochondrial F0 complex, subunit B1	SYBR-green	Thermo Fisher [2]
Bmp4	Bone morphogenetic protein 4	Taqman	Thermo Fisher (Mm00432087_m1)
Cebpa	CCAAT Enhancer Binding Protein Alpha	SYBR-green	Thermo Fisher [1]
Ctnnb1	Catenin (cadherin associated), beta 1	Taqman	Thermo Fisher (Mm00483039_m1)
Cxcl12	C-X-C Motif Chemokine Ligand 12	SYBR-green	Thermo Fisher [1]
Cxcl12	Chemokine (C-X-C motif) ligand 12	Taqman	Thermo Fisher (Mm00445553_m1)
Cxcr4	Chemokine (C-X-C motif) receptor 4	Taqman	Thermo Fisher (Mm01292123_m1)
Fabp4	Fatty acid binding protein 4, adipocyte	SYBR-green	Qiagen (QT00091532)
Gapdh	Glyceraldehyde-3-phosphate dehydrogenase	Taqman	Thermo Fisher (Mm99999915_g1)
Icam1	Intercellular adhesion molecule 1 (also known as CD54)	SYBR-green	QT00155078
Kit	Kit oncogene	Taqman	Thermo Fisher (Mm00445212_m1)
Kitl	Kit ligand	Taqman	Thermo Fisher (Mm00442972_m1)
Lepr	Leptin receptor	SYBR-green	Qiagen (QT00154133)
Lpl	Lipoprotein lipase	SYBR-green	Thermo Fisher [1]
Ly6a	Lymphocyte antigen 6 complex; also known as Sca1 (stem cell antigen-1)	Taqman	Thermo Fisher (Mm00726565_s1)
Map3k7	mitogen-activated protein kinase kinase 7 (TAK1)	Taqman	Thermo Fisher (Mm00554514_m1)
Nanog	Nanog homeobox	Taqman	Thermo Fisher (Mm02019550_s1)
Pgk1	Phosphoglycerate kinase 1	SYBR-green	Thermo Fisher [2,3]
Pou5f1	POU domain, class 5, transcription factor 1; also known as Oct4 (Octamer-binding protein 4)	Taqman	Thermo Fisher (Mm03053917_g1)
Pparg	Peroxisome proliferator activated receptor gamma	SYBR-green	Qiagen (QT00100296)
Ptprc	protein tyrosine phosphatase, receptor type, C; Cd45	Taqman	Thermo Fisher (Mm01293577_m1)
Runx2	Runt related transcription factor 2	Taqman	Thermo Fisher (Mm00501584_m1)
Smad2	SMAD family member 2	Taqman	Thermo Fisher (Mm00487530_m1)
Smad3	SMAD family member 3	Taqman	Thermo Fisher (Mm01170760_m1)

Smad6	SMAD family member 6	Taqman	Thermo Fisher (Mm00484738_m1)
Sox2	SRY (sex determining region Y)-box 2	Taqman	Thermo Fisher (Mm03053810_s1)
Sp7	Sp7 transcription factor	SYBR-green	Qiagen (QT00293181)
Sp7	Sp7 transcription factor	Taqman	Thermo Fisher (Mm04933803_m1)
Tgfb1	Transforming growth factor, beta 1	SYBR-green	Qiagen (QT00145250)
Tgfb1	Transforming growth factor, beta 1	Taqman	Thermo Fisher (Mm01178820_m1)
Tgfbr1	Transforming growth factor, beta receptor 1	SYBR-green	Qiagen (QT00135828)
Tgfbr1	Transforming growth factor, beta receptor 1	Taqman	Thermo Fisher (Mm00436964_m1)
Tgfbr2	Transforming growth factor, beta receptor 2	SYBR-green	Qiagen (QT00135646)
Tgfbr2	Transforming growth factor, beta receptor 2	Taqman	Thermo Fisher (Mm00436977_m1)
Vcam1	Vascular cell adhesion protein 1	SYBR-green	Qiagen (QT00128793)
Wnt11	wingless-type MMTV integration site family, member 11	Taqman	Thermo Fisher (Mm00437328_m1)

Supplementary Table S8: qPCR primers for human genes.

Gene	Gene description	Chemistry	Qiagen Cat#
ACTB	Actin, beta (housekeeping gene)	SYBR-green	PPH00073G; QT01680476
ACTB	Actin, beta (housekeeping gene)	Taqman	Hs01060665_g1
ACSL1	Acyl-CoA synthetase long chain family member 1	Taqman	Hs00960561_m1
B2M	Beta-2-microglobulin (housekeeping gene)	SYBR-green	PPH01094E
BGLAP	Bone gamma-carboxyglutamate protein	SYBR-green	PPH01898A; QT00232771
BMPR1A	Bone morphogenetic protein receptor, type IA	SYBR-green	PPH01929C; QT00085358
BMPR1B	Bone morphogenetic protein receptor, type IB	SYBR-green	PPH01952C; QT00084469
BMPR2	Bone morphogenetic protein receptor, type II (serine/threonine kinase)	SYBR-green	PPH00401B; QT00226065
CCND1	Cyclin D1	SYBR-green	QT00495285
CD36	Cluster determinant 36 (also known as scavenger receptor class B, member 3)	Taqman	Hs00354519_m1
CTNNB1	Catenin (cadherin-associated), beta 1	SYBR-green	PPH00643F; QT00077882
FGF1	Fibroblast growth factor 1 (acidic)	SYBR-green	PPH00067F
FGF2	Fibroblast growth factor 2 (basic)	SYBR-green	PPH00257C
FGFR1	Fibroblast growth factor receptor 1	SYBR-green	PPH00372F
FGFR2	Fibroblast growth factor receptor 2	SYBR-green	PPH00391F
GAPDH	Glyceraldehyde-3-phosphate dehydrogenase (housekeeping gene)	SYBR-green	PPH00150F; QT00079247
HPRT1	Hypoxanthine phosphoribosyltransferase 1 (housekeeping gene)	SYBR-green	PPH01018C
IGF1	Insulin-like growth factor 1 (somatomedin C)	SYBR-green	PPH00167C
IGF1R	Insulin-like growth factor 1 receptor	SYBR-green	PPH00350F
IGF2	Insulin-like growth factor 2 (somatomedin A)	SYBR-green	PPH00168B
LEF1	Lymphoid enhancer-binding factor 1	SYBR-green	PPH02778C; QT00021133
PPARG2	Peroxisome proliferator-activated receptor gamma, isoform 2	SYBR-green	QT00029841
RPLP0	Ribosomal protein, large, P0 (housekeeping gene)	SYBR-green	PPH21138F; QT00075012
RTC	Reverse Transcription Control	SYBR-green	PPX63340A
RUNX2	Runt-related transcription factor 2	SYBR-green	PPH01897C; QT00020517
SLC27A1	Solute carrier family 27 member 1 (also known as fatty acid transport protein 1, *FATP1*)	Taqman	Hs01587911_m1
SMAD1	SMAD family member 1	SYBR-green	PPH01174A
SMAD2	SMAD family member 2	SYBR-green	PPH01949F
SMAD3	SMAD family member 3	SYBR-green	PPH01921C
SMAD4	SMAD family member 4	SYBR-green	PPH00134C
SMAD5	SMAD family member 5	SYBR-green	PPH01940C
SP7	Sp7 transcription factor	SYBR-green	PPH00705A; QT00213514
TCF7	Transcription factor 7 (T-cell specific, HMG-box)	SYBR-green	PPH02757B; QT00095410

TCF7L1	Transcription factor 7-like 1 (T-cell specific, HMG-box)	SYBR-green	PPH02745B; QT00091735
TCF7L2	Transcription factor 7-like 2 (T-cell specific, HMG-box)	SYBR-green	PPH06890C; QT00071120
TGFB1	Transforming growth factor, beta 1	SYBR-green	PPH00508A; QT00000728
TGFB2	Transforming growth factor, beta 2	SYBR-green	PPH00524B
TGFB3	Transforming growth factor, beta 3	SYBR-green	PPH00531F
TGFBR1	Transforming growth factor, beta receptor 1	SYBR-green	PPH00237C; QT00083412
TGFBR2	Transforming growth factor, beta receptor II (70/80kDa)	SYBR-green	PPH00339C
WISP1	WNT1 inducible signaling pathway protein 1	SYBR-green	QT00079492

Supplementary Table S9: Antibodies and conditions for mouse bone tissue staining.

Target	Alternative names	Clone	Source	Catalogue number	Host Species	Antigen Retrieval Buffer	Figure #
CD45	Ly5, LCA	Poly	Thermo Fisher	20103-1-AP	Rabbit	TRIS/EDTA	S11
CXCL12	SDF-1	Poly	Thermo Fisher	14-7992-81	Rabbit	TRIS/EDTA	S16
INS		Poly	Thermo Fisher	15848-1-AP	Rabbit	Citrate/EDTA	S2
LEPR		Poly	R&D Systems	AF497	Mouse	Citrate/EDTA	S18
PLIN1		Poly	Thermo Fisher	PA5-55046	Rabbit	TRIS/EDTA	1, S6
TGFB1		Poly	Proteintech	21898-1-AP	Rabbit	TRIS/EDTA	3
SCA1	Ly6A	Mono	Abcam	ab109211	Rabbit	Citrate/EDTA	2
SOX2		Poly	Thermo Fisher	48-1400	Rabbit	TRIS/EDTA	2

Supplementary Table S10: Exposure times of various test agents in cell culture studies.

Illustration #	Test agents	Exposure duration
Figure 5	Adipogenesis inducing media (ADP); recombinant human transforming growth factor beta 1 (TGFB1); 25 mM glucose (HG)	72 hours
Figure 6	Adipogenesis inducing media (ADP); recombinant human transforming growth factor beta 1 (TGFB1); signaling inhibitors (ALK5, TAK1, JNK, SMAD3, P38, ERK, PKC, PI3K)	72 hours
Figure 7; Table S7-S11	Adipogenesis inducing media (ADP); recombinant human transforming growth factor beta 1 (TGFB1); TAK1 inhibitor	48 hours
Figure S10	Insulin-deficient adipogenesis induction media (idADP); Linoleic-Oleic acid (LIN/OL); 25 mM glucose (HG)	7 days
Figure S20	25 mM glucose (HG)	21 days
Figure S21	Adipogenesis inducing media (ADP); recombinant human transforming growth factor beta 1 (TGFB1); 25 mM glucose (HG); TALK1 inhibitor	72 hours
Figure S22	Osteogenesis inducing media (OST); recombinant human transforming growth factor beta 1 (TGFB1)	9 days

Supplementary Table S11: Pharmacological inhibitors used in cell culture studies.

Reagent	Target protein	Acronym	Concentrations tested	Source (Catalogue #)
GW 788388	ALK5	ALK5i	1, 10, 50 µmol/L	Tocris Bioscience (3264)
Chelerythrine Chloride	PKC	PKCi	0.1, 1, 5 µmol/L	Cayman Chemical (11314)
SB203580	p38 MAPK	p38i	1, 10, 50 µmol/L	Millipore (559395)
(5Z)-7-Oxozeaenol	TAK1	TAK1i	1, 10, 50 µmol/L	Cayman Chemical (17459)
JNK inhibitor XVI	JNK	JNKi	1, 10, 50 µmol/L	Cayman Chemical (18096)
SIS3	SMAD3	SMAD3i	1, 10, 50 µmol/L	Cayman Chemical (15945)
PI 828	PI3K	PI3Ki	1, 10, 50 µmol/L	Tocris Bioscience (2814)
PD98059	MEK1,2	MEKi	1, 10, 50 µmol/L	STEMCELL Technologies (72172)

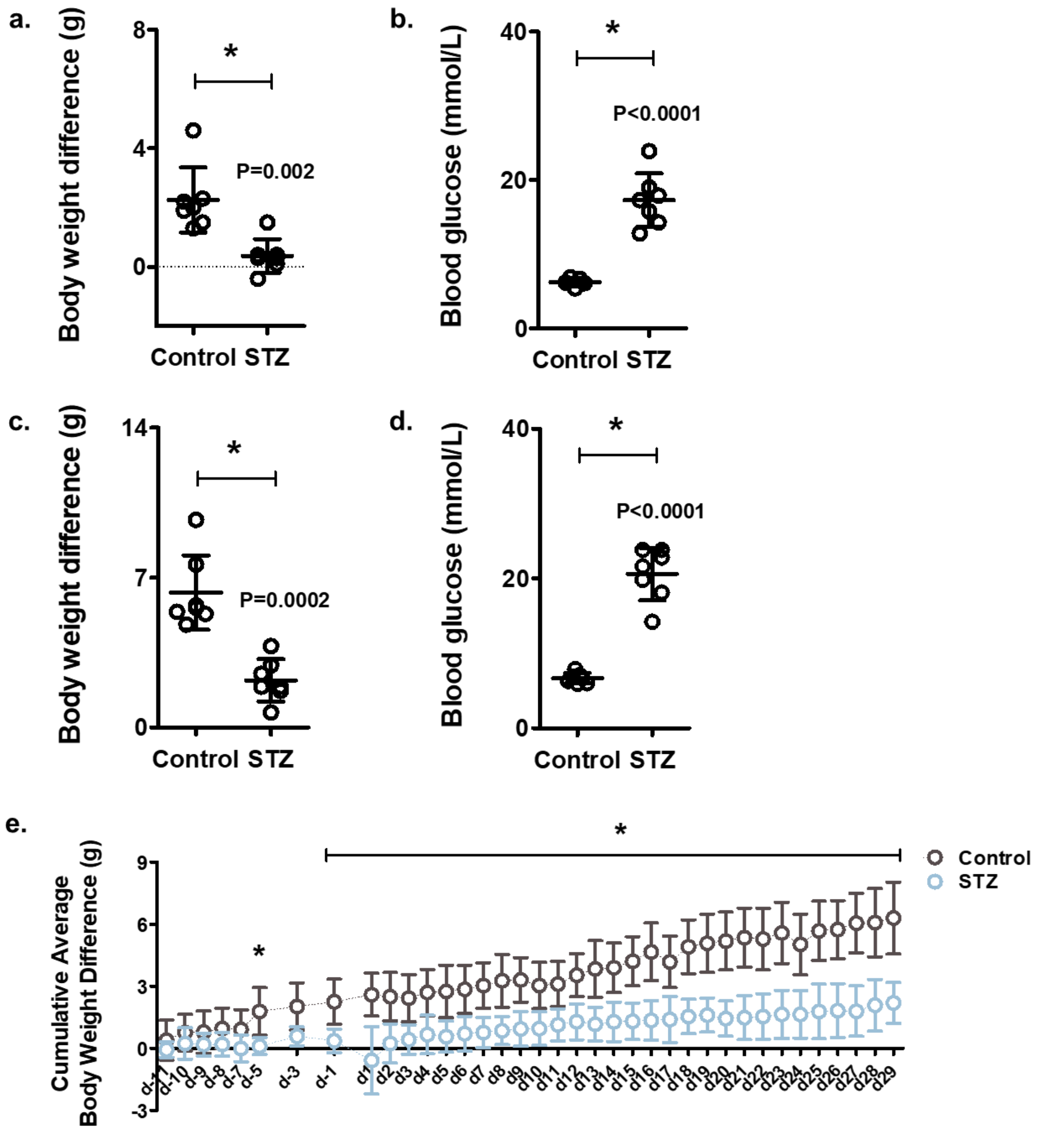

Supplementary Figure S1: ***Streptozotocin-induced diabetes causes impaired weight gain in mice at 1 month.***

Diabetes was induced in C57BL/6 mice with daily intraperitoneal injections of streptozotocin (STZ; 50 mg/kg) for 5 consecutive days. Non-diabetic control mice received an equal volume of citrate buffer. Blood glucose levels were checked 1 week after the last STZ injection to confirm hyperglycemia. (a) The body weight difference was calculated 1 week after the last STZ injection. [Mean ± SD; n = 7; each data point represents a mouse; two-tailed student's t-test: * $p<0.05$]. (b) Non-fasting blood glucose levels in mice measured 1 week after the last STZ injection [Mean ± SD; n = 5-7; each data point represents a mouse; two-tailed student's t-test: * $p<0.05$]. (c) Body weight difference at the conclusion of the study (d29) [Mean ± SD; n = 7; each data point represents a mouse; two-tailed student's t-test: * $p<0.05$]. (d) Non-fasting blood glucose levels measured at the conclusion of the study (d28) [Mean ± SD; n = 7; each data point represents a mouse; two-tailed student's t-test: * $p<0.05$]. (e) Cumulative average body weight

difference from d-11 to d29 comparing non-diabetic Control and STZ mice [Mean ± SD; n = 7; Two-way ANOVA followed by Bonferroni post hoc analysis: * $p<0.05$].

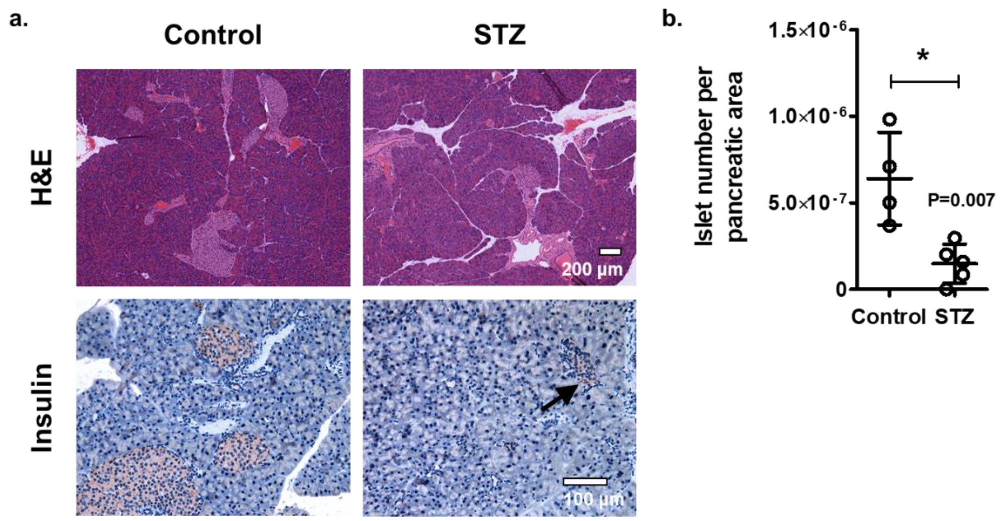

Supplementary Figure S2: ***Diminished pancreatic islets in streptozotocin-induced diabetic mice at 1 month.***

Pancreata were harvested from C57BL/6 male mice, 1 month after administration of streptozotocin (STZ; 50 mg/kg) or citrate buffer control (Control). (a) Representative images showing H&E-stained sections (upper panel) and insulin immunoreactivity (lower panel). Insulin reactivity was detected by chromogen staining (arrow in STZ group indicating positive reactivity in a diminished islet) [scale bar = 200 µm for H&E and 100 µm for insulin staining]. (b) The number of pancreatic islets (insulin-positive islets; manual count) per pancreatic area (determined by *QuPath*) is shown [Mean ± SD; n = 4 control and 5 STZ; each data point represents a mouse; two-tailed student's t-test: * $p<0.05$].

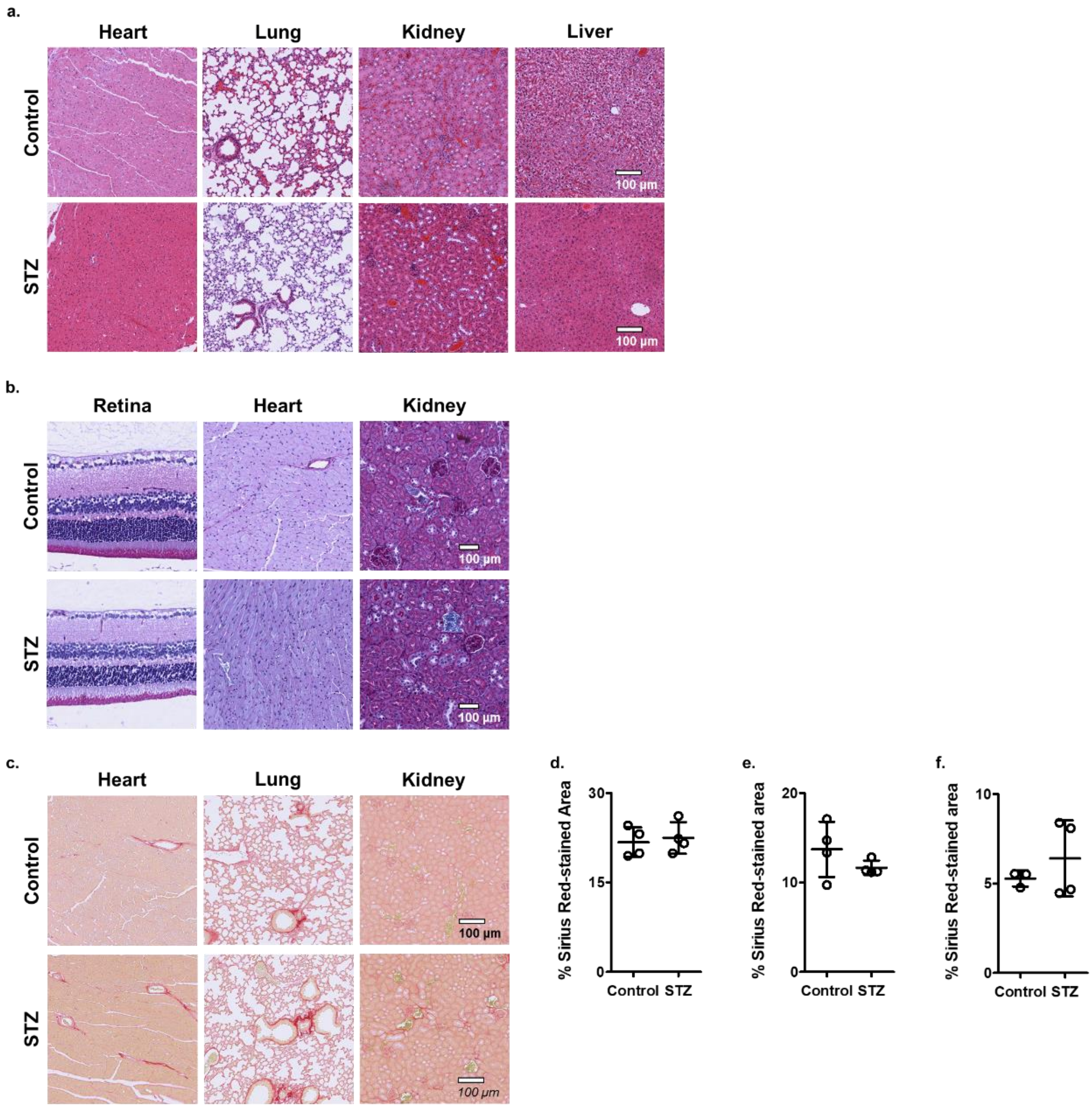

Supplementary Figure S3: ***Histological analyses of STZ-induced diabetic mice at 1 month.***

Tissues were harvested from C57BL/6 male mice, 1 month after administration of streptozotocin (STZ; 50 mg/kg) or citrate buffer control (Control). (a) Representative H&E-stained images of heart, lung, kidney, and liver tissue of mice [scale bar = 100 μm]. (b) Periodic Acid-Schiff (PAS)-stained images of retina, heart, and kidney tissues [scale bar = 100 μm]. (c) Picro-Sirius red-stained tissues of mice [scale bar = 100 μm]. (d-f) Quantitative assessment of Picro-Sirius red-stained area of the heart (d), lung (e), and kidney (f). Stained areas were determined with

ImageJ [Mean ± SD; n = 4 controls and 4 STZ in panels d and e, 3 controls and 4 STZ in panel f; each data point represents a mouse; two-tailed student’s t-test: * $p<0.05$].

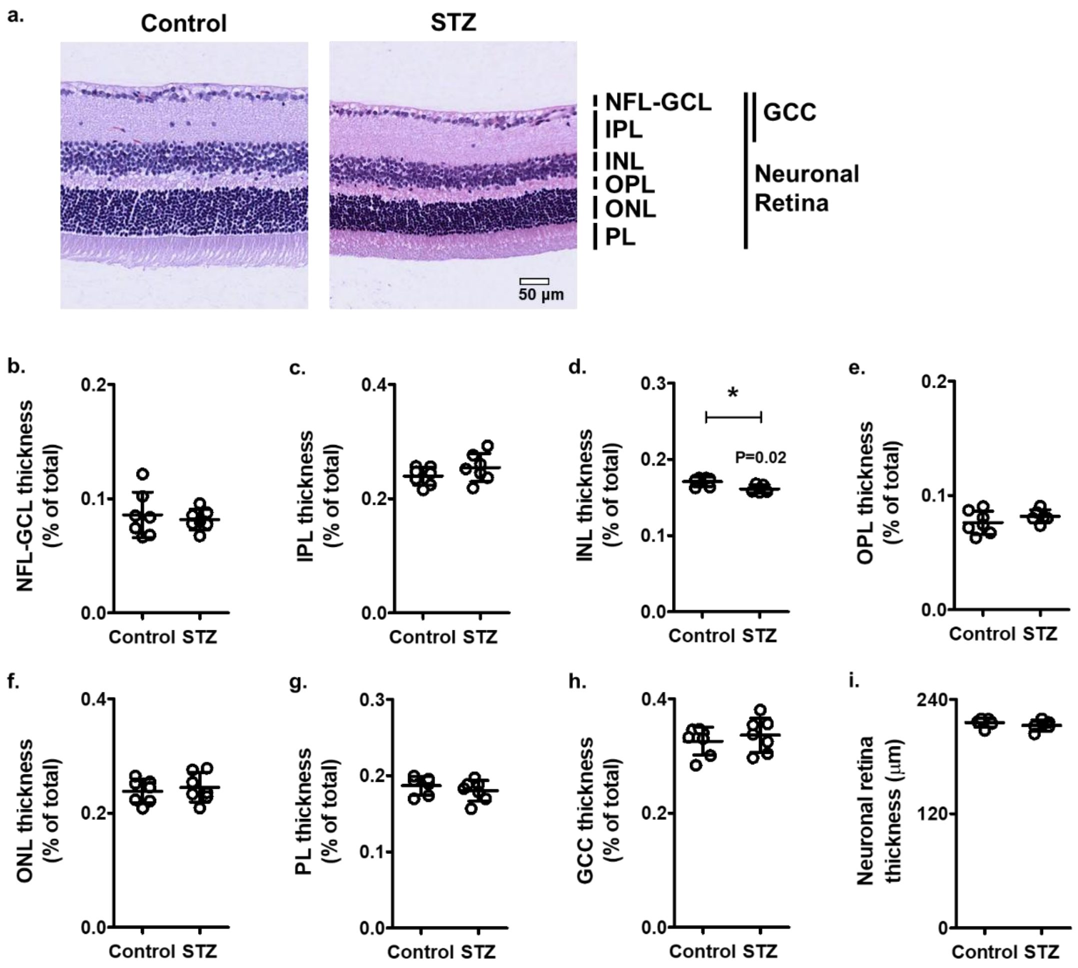

Supplementary Figure S4: ***Reduced inner nuclear layer thickness of the retina in mice after 1 month of streptozotocin-induced diabetes.***

Eyes were harvested from C57BL/6 male mice, 1 month after administration of streptozotocin (STZ; 50 mg/kg) or citrate buffer control (Control). (a) Representative H&E-stained sections of the retina in C57BL/6 mice [scale bar = 50 μm]. (b-i) The thickness of the retinal layer was measured as a percentage of the neuronal retina thickness in the control and diabetic mouse [Mean ± SD; n = 6-8; each data point represents a mouse; two-tailed student's t-test: * $p<0.05$]. Abbreviations: NFL, nerve fiber layer; GCL, ganglion cell layer; IPL, inner plexiform layer; INL, inner nuclear layer; OPL, outer plexiform layer; ONL, outer nuclear layer; PL, photoreceptor layer; and GCC, ganglion cell complex.

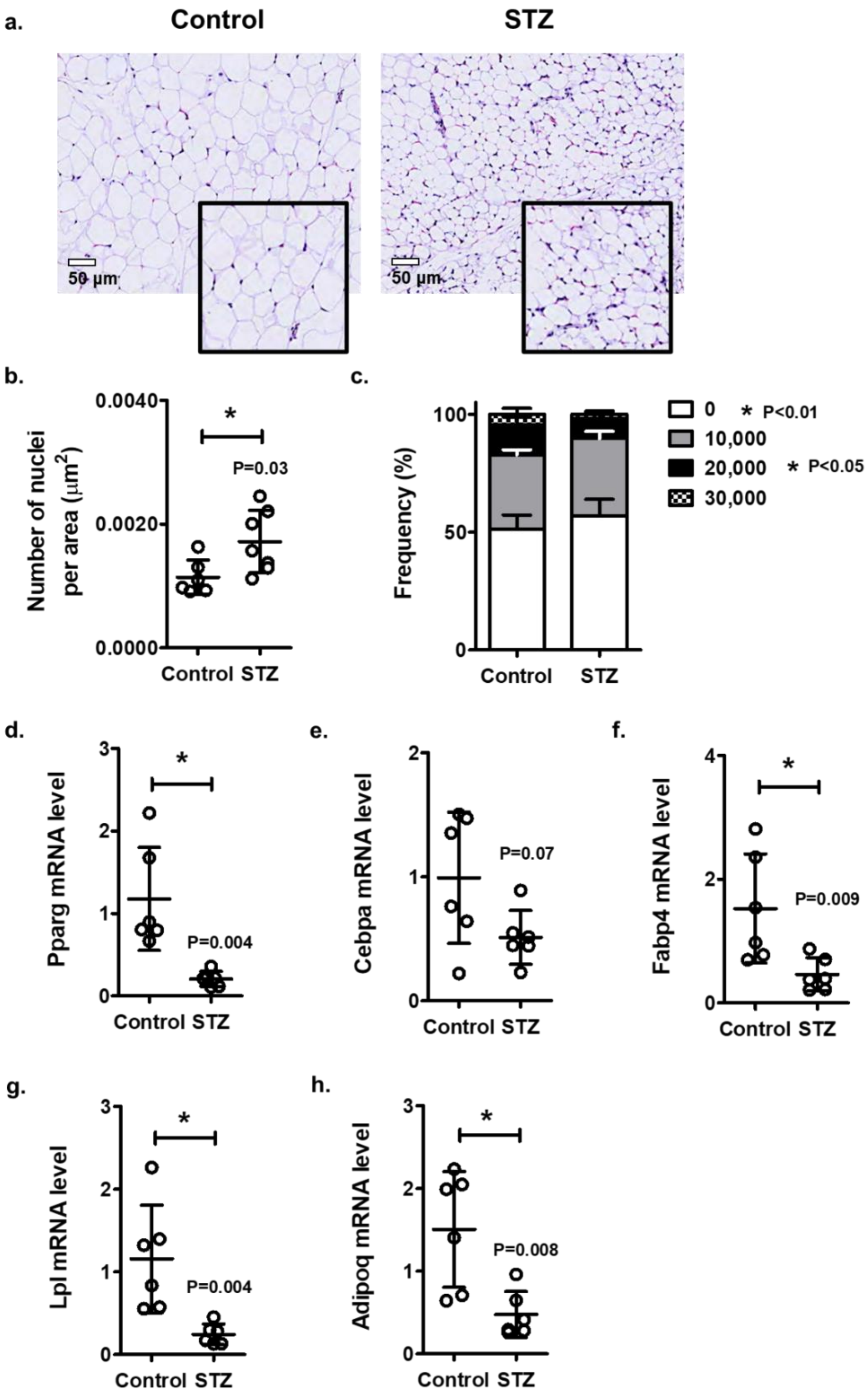

Supplementary Figure S5: ***Cellular hyperplasia in white adipose tissue of streptozotocin-induced diabetic mice at 1 month.***

Epididymal fat was harvested from C57BL/6 male mice, 1 month after administration of streptozotocin (STZ; 50 mg/kg) or citrate buffer control (Control). (a) Representative H&E-

stained sections [scale bar = 50 μm]. Inserts showing higher magnification. (b) The number of nuclei per tissue area was measured by *QuPath* [Mean ± SD; n = 6 control and 7 STZ; each data point represents a mouse; two-tailed student's t-test: * p<0.05]. (c) Frequency of the area of the individual adipocytes of different sizes found in epididymal adipose tissue. Frequency was determined by *Adiposoft* [Mean ± SD; n = 6 control and 7 STZ; two-way ANOVA followed by Bonferroni post hoc analysis: * p<005]. (d-h) mRNA levels of *Pparg* (d), *Cebpa* (e), *Fabp4* (f), *Lpl* (g), and *Adipoq* (h) in epididymal adipose tissue [Data normalized to *Actb*; Mean ± SD; n = 6 control and 7 STZ; each data point represents a mouse; two-tailed student's t-test: * p<0.05].

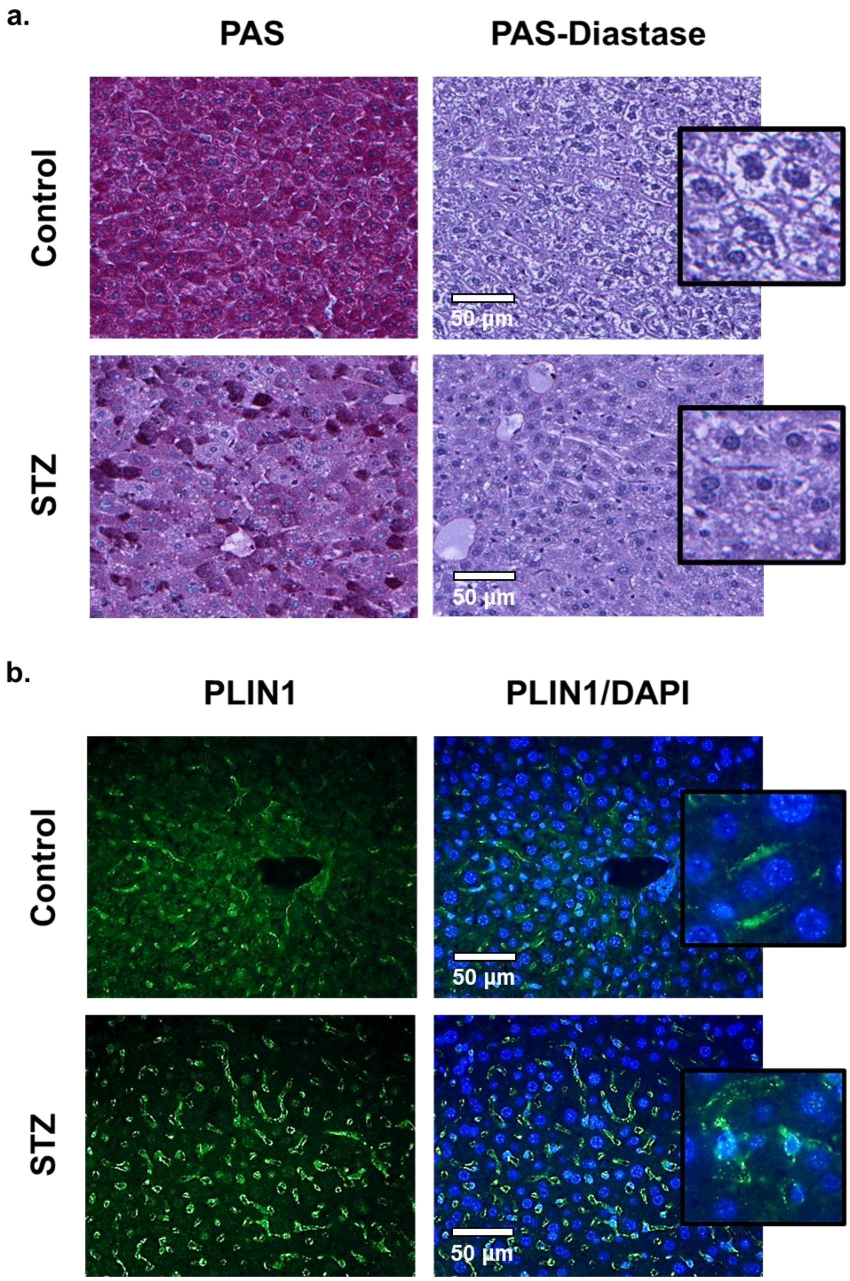

Supplementary Figure S6: ***Increased lipid accumulation in the liver of streptozotocin-induced diabetic mice at 1 month.***

Liver tissues were harvested from C57BL/6 male mice, 1 month after administration of streptozotocin (STZ; 50 mg/kg) or citrate buffer control (Control). (a) Representative images of the liver showing periodic Acid-Schiff (PAS) staining performed with or without the combination of diastase (α-amylase) [scale bar = 50 μm]. Inserts showing higher magnification. (b) Immunofluorescence staining of liver tissues for perilipin-1 (PLIN1; green). Sections were counterstained with DAPI (blue) [scale bar = 50 μm]. Inserts showing higher magnification.

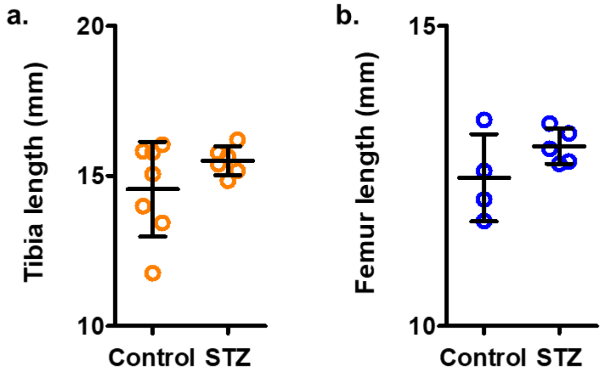

Supplementary Figure S7: ***Changes in femur and tibia length after 1 month of diabetes.***

(a,b) Lengths of the tibia (a) and femur (b) were measured by *QuPath* in control and streptozotocin (STZ)-induced diabetic mice after 1 month [Mean ± SD; n=7 control and 6 STZ in panel a, 4 control and 5 STZ in panel b; each data point represents a mouse; two-tailed student's t-test: * p<0.05].

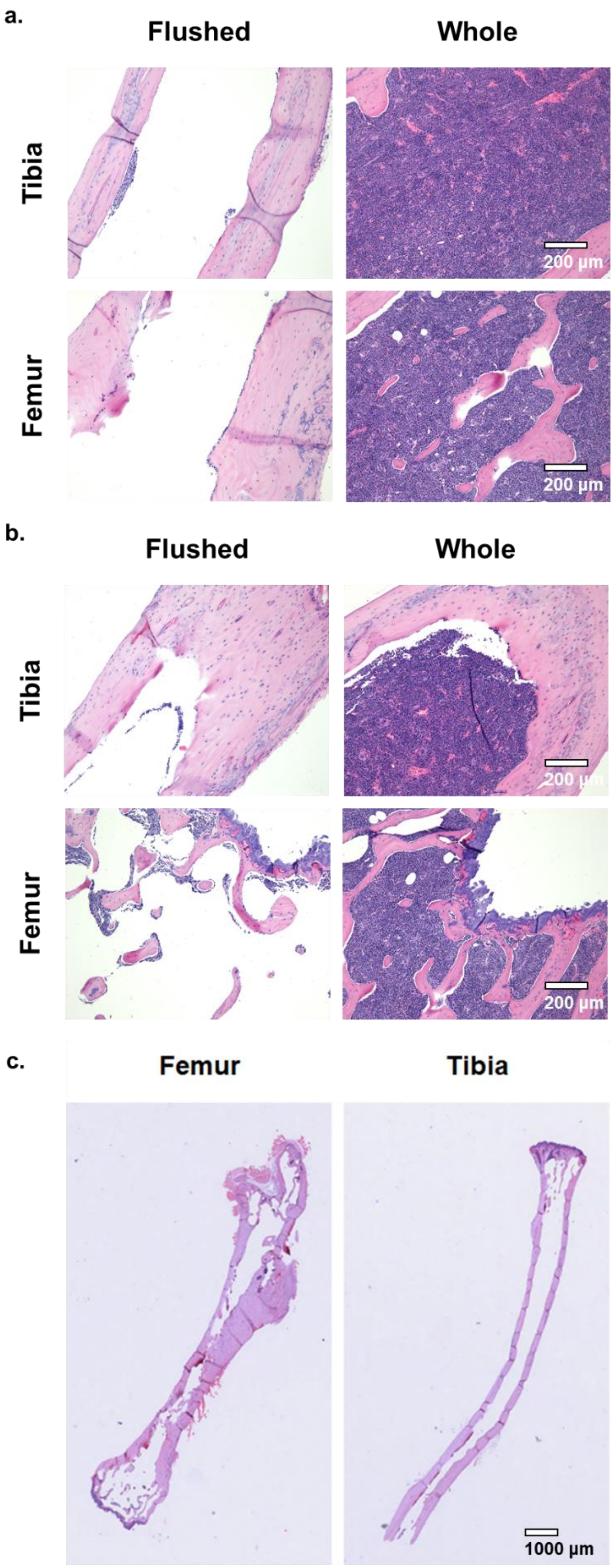
a.
Flushed
Whole
Tibia
Femur
200 µm
200 µm
b.
Flushed
Whole
Tibia
Femur
200 µm
200 µm
c.
Femur
Tibia
1000 µm

Supplementary Figure S8: ***Quality control of the bone marrow flush method.***

Representative hematoxylin and eosin (H&E)-stained sections of mouse tibia and femur after marrow flush (‘flushed’) or without flush (‘whole’). Figure showing areas of the bone shaft (a) and distal ends (b) [scale bar = 200 μm]. (c) Whole femur and tibia after flush [scale bar = 1000 μm].

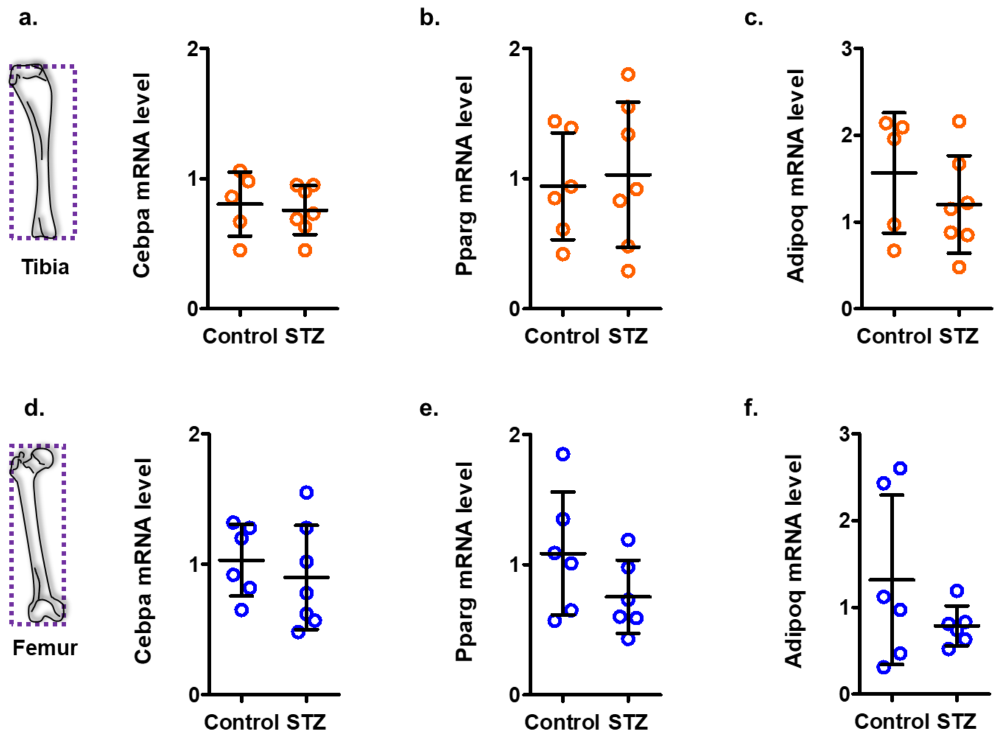

Supplementary Figure S9: ***Expression of adipogenesis-associated genes shows no difference in the bone marrow after 1 month of streptozotocin-induced diabetes.***

Tibiae (a-c) and femurs (d-f) of non-diabetic control and streptozotocin (STZ)-induced diabetic mice, 1 month after the onset of diabetes, were flushed to isolate marrow cells for mRNA analyses. Levels of adipogenesis-associated genes were measured [For panels (a,d), data normalized to *Actb* and *Gapdh*; for panels (b,c,e,f), data normalized to *Actb, Atp5f1,* and *Pgk1*; Mean ± SD; n = 5 control and 7 STZ in panel a, 6 control and 6 STZ in panel b, 5 control and 7 STZ in panel c, 6 control and 7 STZ in panel d, 6 control and 6 STZ in panels e and f; each data point represents a mouse; two-tailed student's t-test: * p<0.05].

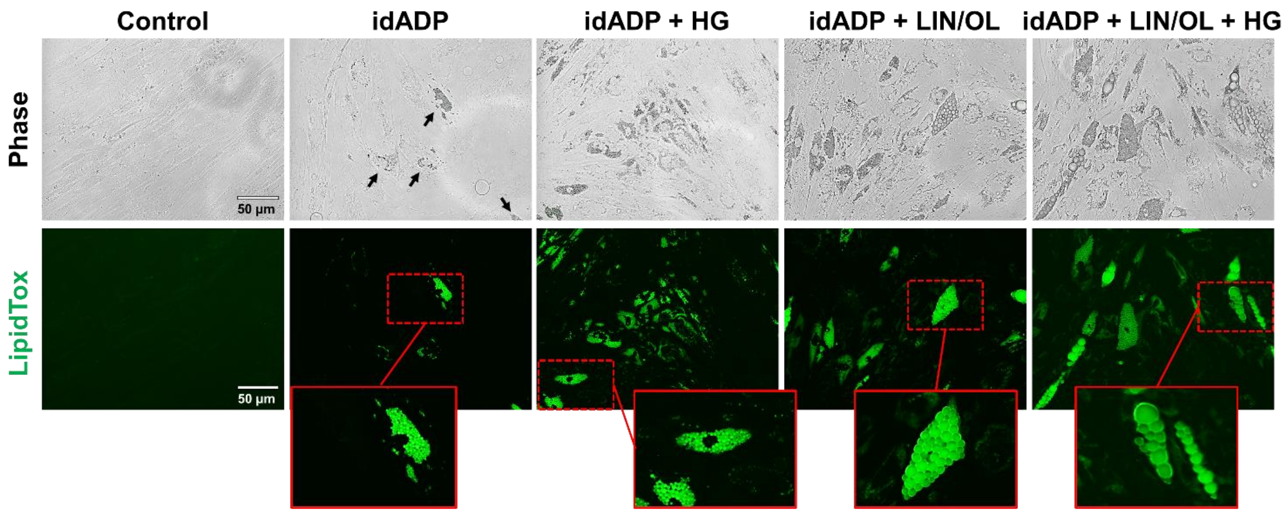

Supplementary Figure S10: Insulin-independent lipid accumulation in bone marrow-derived mesenchymal progenitor cells.

Human bone marrow-derived progenitor cells (bm-MPCs) were exposed to an insulin-deficient adipogenesis induction media (idADP; Table S5) for 7 days. idADP was supplemented with either 25 mM glucose (high glucose; HG), linoleic acid-oleic acid mixture (LIN/OL), or a combination of HG and LIN/OL. Upper panel showing phase contrast images of cells [arrows point to lipid droplets in cells exposed to idADP]. Lower panel showing cells stained with LipidTOX (green) to detect intracellular lipid accumulation. Images are representative of 4 experimental replicates [scale bar = 50 µm]. Inserts showing high-power images.

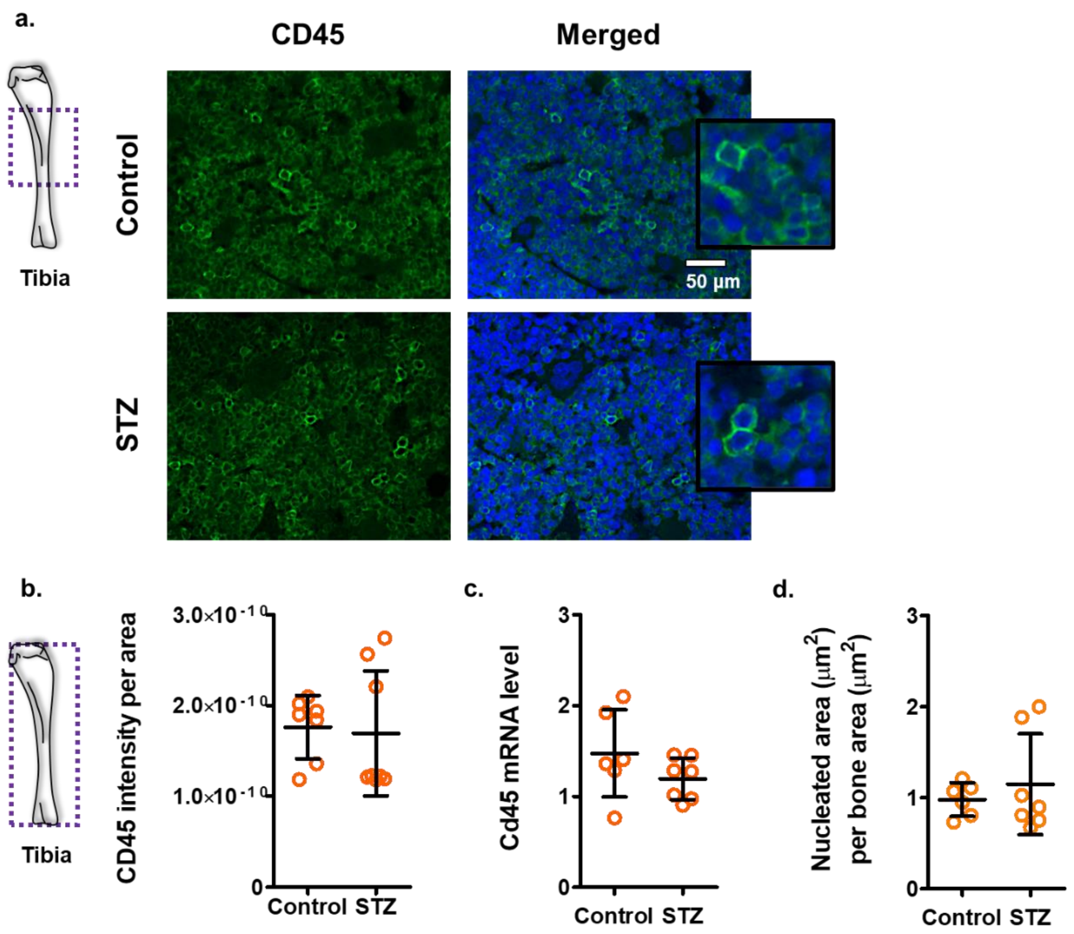

Supplementary Figure S11: ***Hematopoietic area in the tibia of streptozotocin-induced diabetic mice at 1 month is not altered.***

(a) Immunofluorescence staining of the tibiae of control and diabetic (STZ; 1 month) mice for CD45 (green). Sections were counterstained with DAPI (blue) [scale bar = 50 µm]. Inserts showing higher magnification. (b) Quantification of CD45 intensity per area, as determined by *ImageJ* [Mean ± SD; n = 7 control and 7 STZ; each data point represents an independent sample; two-tailed student's t-test: * p<0.05]. (c) *Cd45* (*Ptprc*) mRNA levels in tibia flush samples [Data normalized to *Actb* and *Gapdh*; Mean ± SD; n = 6 control and 7 STZ; each data point represents a mouse; two-tailed student's t-test: * p<0.05]. (d) Nucleated area per bone area measured using *MarrowQuant* [Mean ± SD; n = 6 control and 7 STZ; each data point represents a mouse; two-tailed student's t-test: * p<0.05].

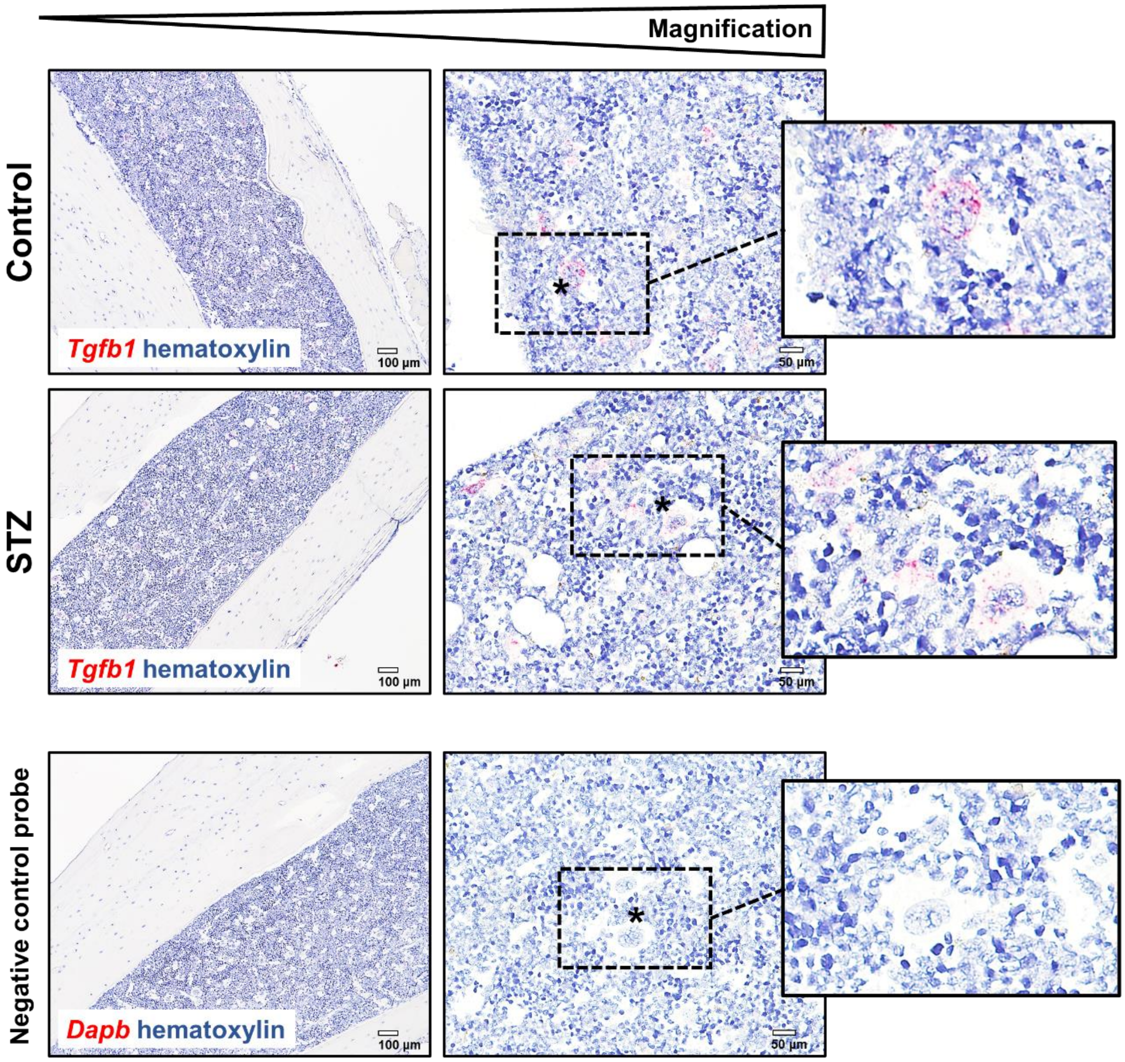

Supplementary Figure S12: ***Localization of Tgfb1 mRNA in the tibia of non-diabetic and streptozotocin-induced diabetic mice.***

Representative images of tibiae of control and diabetic (STZ; 1 month) mice for *Tgfb1* (red). Sections were counterstained with hematoxylin (blue). Bacillus subtilis dihydrodipicolinate reductase (*dapb*) probe was used as a negative control [scale bar = 100 µm for low power images on left, 50 µm for high power images on right]. Inserts showing higher magnification. Asterisks highlighting select megakaryocytes.

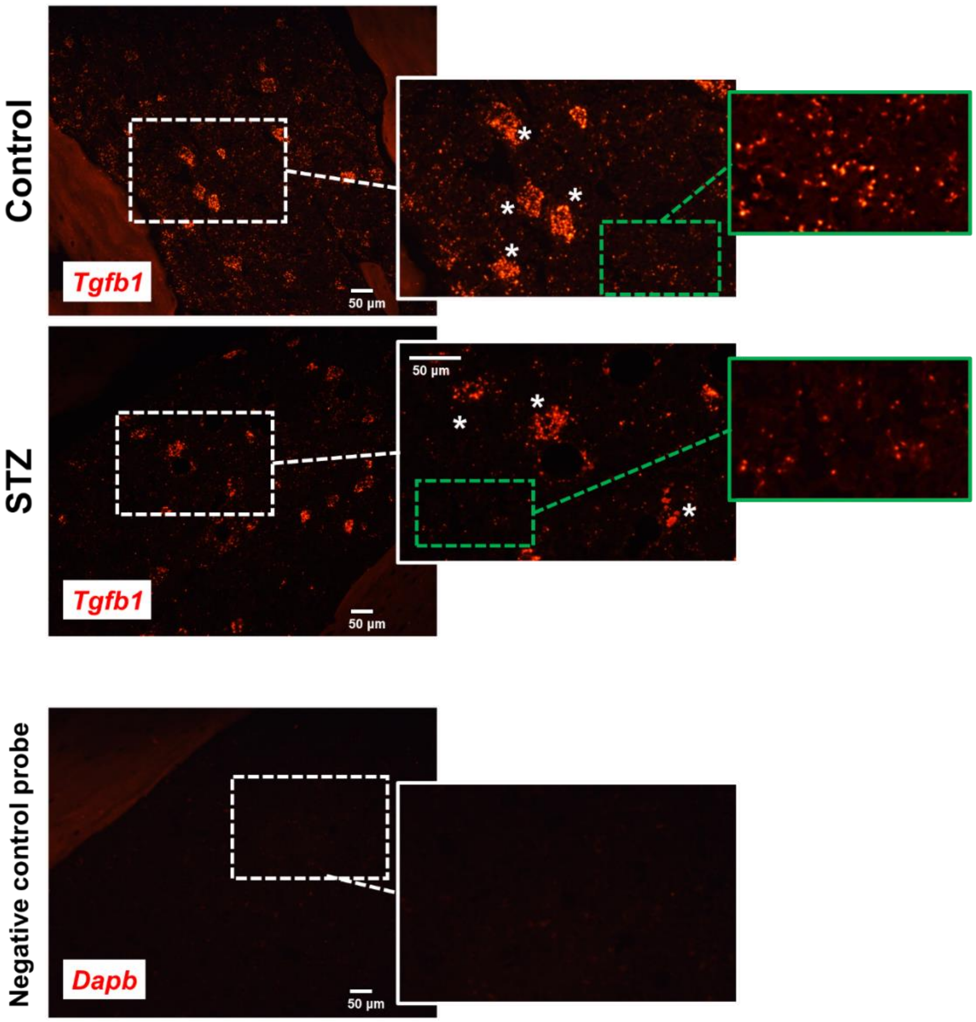

Supplementary Figure S13: ***Fluorescence images of Tgfb1 in situ hybridization.***

Representative images of tibiae of control and diabetic (STZ; 1 month) mice for *Tgfb1* (red) [scale bar = 50 µm]. Bacillus subtilis dihydrodipicolinate reductase (*dapb*) probe was used as a negative control. Probes were detected RNAscope 2.5 HD Assay Red (chromogen-based; Red). Fluorescence images were taken using TRITC filter. RNAscope 2.5 Red Reagent is naturally fluorescent around 570 nm (TRITC/CY3). Inserts showing higher magnification. Asterisks highlighting select megakaryocytes. Second-level inserts (in green) show that most bone marrow cells express *Tgfb1*.

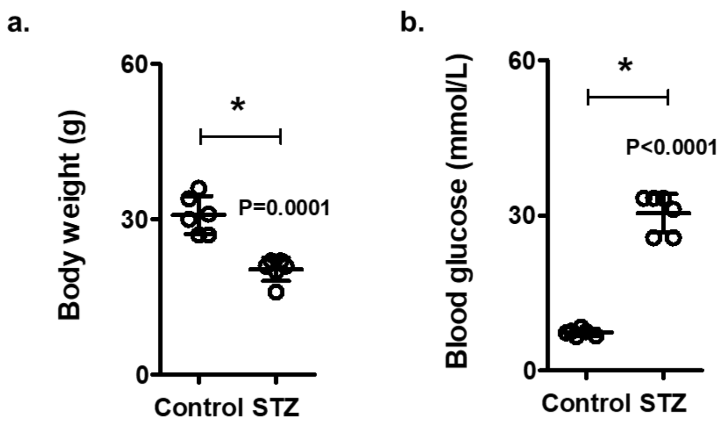

Supplementary Figure S14: ***Impaired weight gain and hyperglycemia in diabetic mice at 2 months.***

C57BL/6 male mice received streptozotocin (STZ; 50 mg/kg) or citrate buffer (non-diabetic controls). (a) Body weights of mice at 2 months of follow-up [Mean ± SD; n = 6; each data point represents a mouse; two-tailed student's t-test: * p<0.05]. (b) Non-fasting blood glucose levels in mice, 2 months after the onset of diabetes [Mean ± SD; n = 6; each data point represents a mouse; two-tailed student's t-test: * p<0.05].

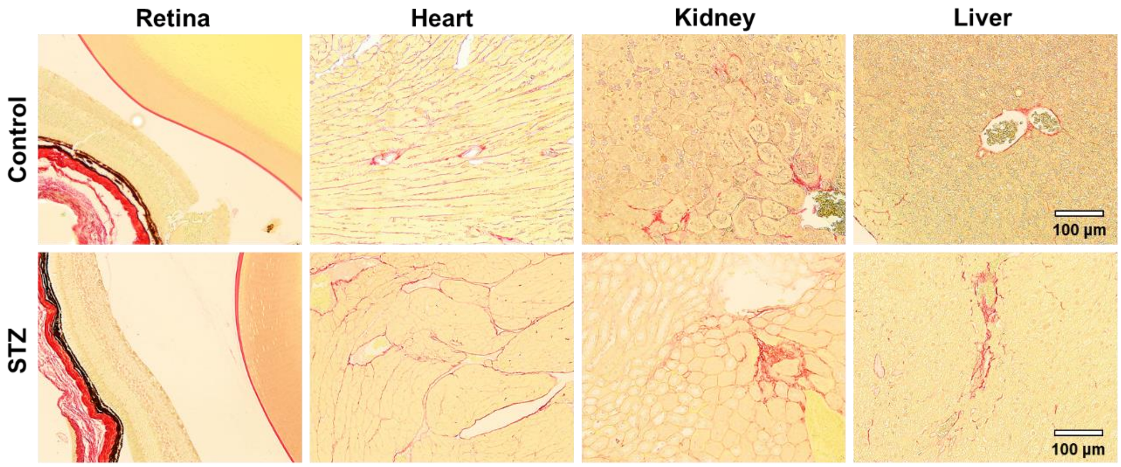

Supplementary Figure S15: ***Picro-Sirius Red staining of tissues harvested from diabetic mice at 2 months.***

Tissues were harvested from C57BL/6 male mice, 2 months after the administration of streptozotocin (STZ; 50 mg/kg) or citrate buffer control (Control). Figure showing Picro-Sirius red-stained tissues of mice [n = 2; scale bar = 100 μm].

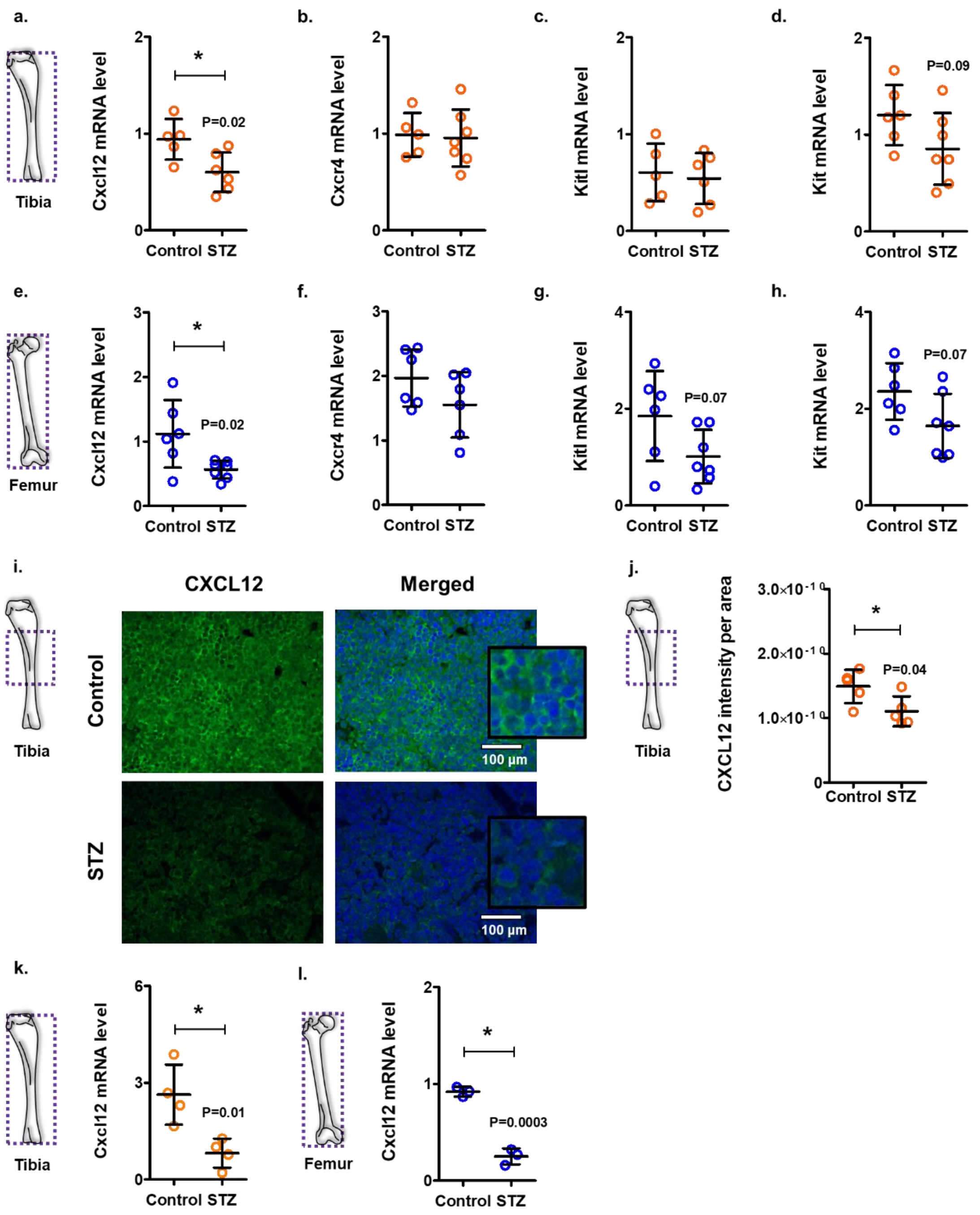

Supplementary Figure S16: ***Diabetes reduces stem cell niche factors in the bone marrow at 1 month.***

(a-h) mRNA levels of *Cxcl12*, *Cxcr4*, *Kitl*, and *Kit* in the tibiae (a-d) and femurs (e-h) of control or diabetic (STZ) mice. Analyses were performed 1 month after the onset of diabetes [For panels (a-d) and (f-h), data normalized to *Actb* and *Gapdh*; for panel (e), data normalized to *Actb*, *Atp5f1*, and *Pgk1*; Mean ± SD; n = 5 control and 6 STZ in panel a, 5 control and 7 STZ in panel b, 5 control and 6 STZ in panel c, 6 control and 7 STZ in panel d, 6 control and 7 STZ in panel e,

6 control and 6 STZ in panel f, 6 control and 7 STZ in panels g and h; each data point represents a mouse; two-tailed student's t-test: * $p<0.05$]. (i) Immunostaining of mouse tibia marrow for CXCL12 (green) after 1 month of STZ-induced diabetes. Sections were counterstained with DAPI (blue) [scale bar = 100 μm]. Inserts showing higher magnification. (j) Quantification of CXCL12 intensity per area, as determined by *ImageJ* [Mean ± SD; n = 4; each data point represents an independent sample; two-tailed student's t-test: * $p<0.05$]. (k, l) mRNA levels of *Cxcl12* were detected in the tibiae (k) and femurs (l) of control or diabetic (STZ) mice after 2 months of diabetes onset [Data normalized to *Actb* and *Gapdh*; Mean ± SD; n = 4 control and 4 STZ in panel k, 3 control and 3 STZ in panel l; each data point represents a mouse; two-tailed student's t-test: * $p<0.05$].

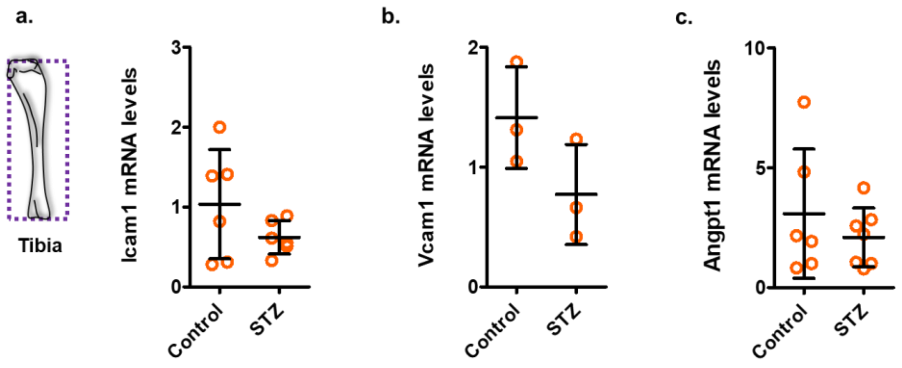

Supplementary Figure S17: ***Streptozotocin-induced diabetic mice show no changes in mRNA levels of other known stem cell niche factors.***

(a-c) mRNA levels of *Icam1*, *Vcam1*, and *Angpt1* in the tibia flush samples from control and streptozotocin (STZ)-induced diabetic mice after 1 month of diabetes onset [For panels a and b, data normalized to *Actb*, *Atp5f1*, and *Pgk1*; for panel c, data normalized to *Actb* and *Gapdh*; Mean ± SD; n = 6 control and 6 STZ in panel a, 3 control and 3 STZ in panel b, 6 control and 7 STZ in panel c; two-tailed student's t-test: * $p<0.05$].

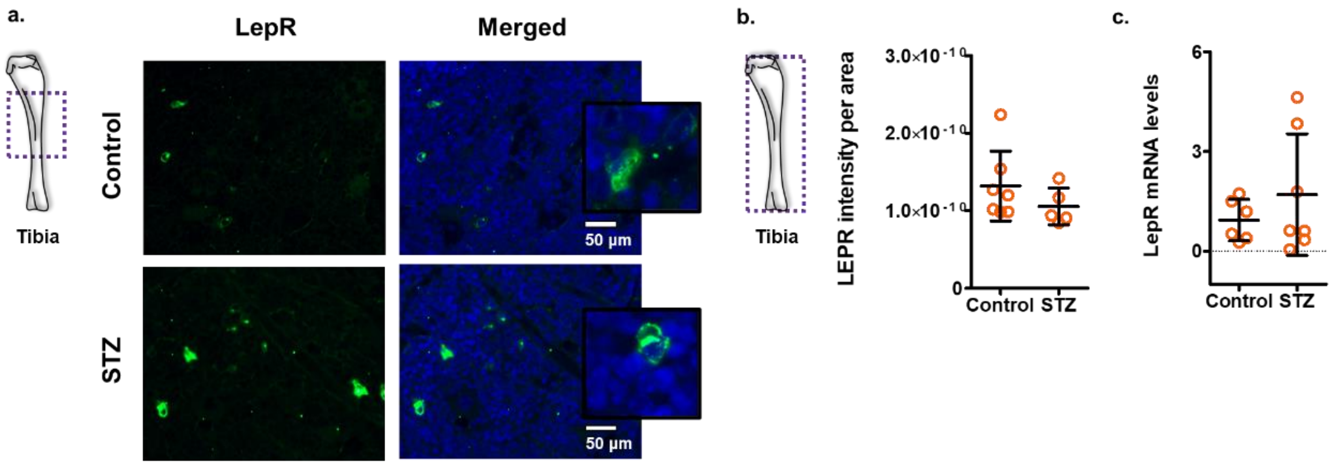

Supplementary Figure S18: ***Streptozotocin-induced diabetic mice show no changes in LEPR-expressing cells at 1 month.***

(a) Representative immunofluorescence staining of LEPR (green) in the shaft of tibia. Sections were counterstained with DAPI (blue) [scale bar = 50 µm]. (b) Quantification of LEPR staining intensity per area, as determined by *ImageJ* [Mean ± SD; n = 6 control and 5 STZ in panel b, 6 control and 7 STZ in panel c; two-tailed student's t-test: * p<0.05]. (c) *Lepr* mRNA levels in the tibia flush samples from control and diabetic (STZ) mice, after 1 month of diabetes onset [Data normalized to *Actb, Atp5f1*, and *Pgk1*; Mean ± SD; n = 5-6; each data point represents a mouse; two-tailed student's t-test: * p<0.05].

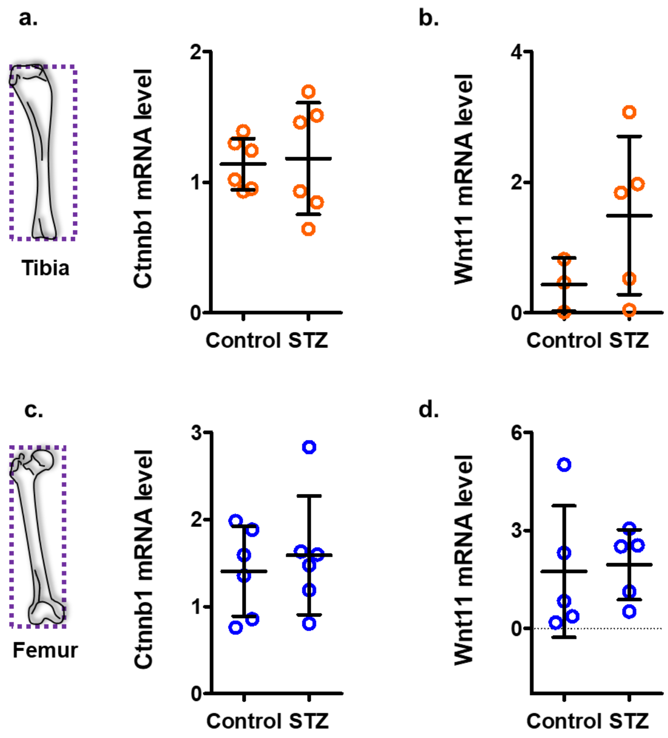

Supplementary Figure S19: ***Wnt signaling pathway genes are unaltered in the marrow of mice after 1 month of diabetes.***

mRNA levels of *Ctnnb1* (catenin beta-1) and *Wnt11* (non-canonical Wnt ligand) were detected in the tibiae (a,b) and femurs (c,d) of control or diabetic (STZ) mice after 1 month of diabetes onset [Data normalized to *Actb* and *Gapdh*; Mean ± SD; n = 6 control and 6 STZ in panel a, 3 control and 5 STZ in panel b, 6 control and 6 STZ in panel c, 5 control and 5 STZ in panel d; each data point represents a mouse; two-tailed student's t-test: * p<0.05].

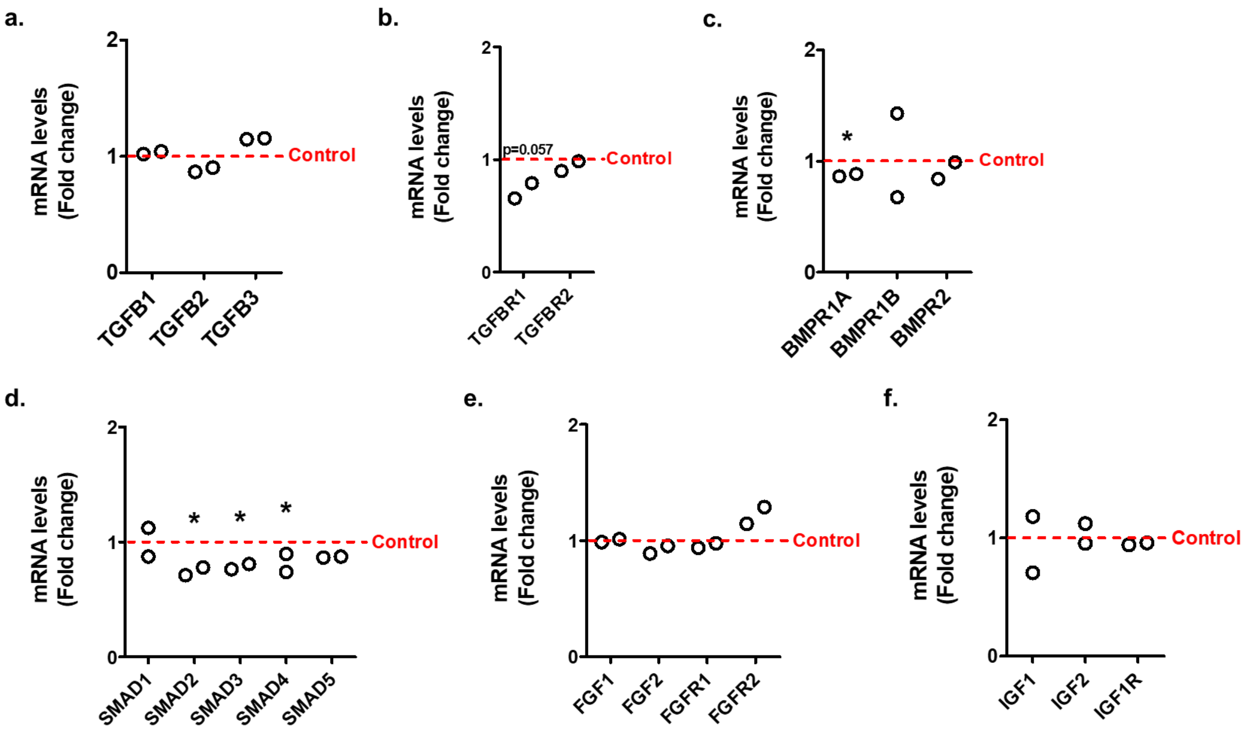

Supplementary Figure S20: ***High levels of glucose reduce TGFB1 pathway genes in bone marrow progenitor cells.***

Human bone marrow-derived progenitor cells were cultured in high glucose (25 mmol/L) for 21 days. Control cells were cultured in media containing 5 mmol/L glucose. Media was changed every other day. mRNA levels of TGFB ligands (a), TGFB receptors (b), BMP receptors (c), SMADs (d), fibroblast growth factor (FGF) signaling pathway (e), and insulin-like growth factor (IGF) signaling pathway (f) were measured. Red dashed line showing mean levels in control cells [Data normalized to *ACTB*, *B2M*, *GAPDH*, *HPRT1*, and *RPLP0*; n = 2; each data point represents an independent sample; two-tailed student's t-test: * $p<0.05$].

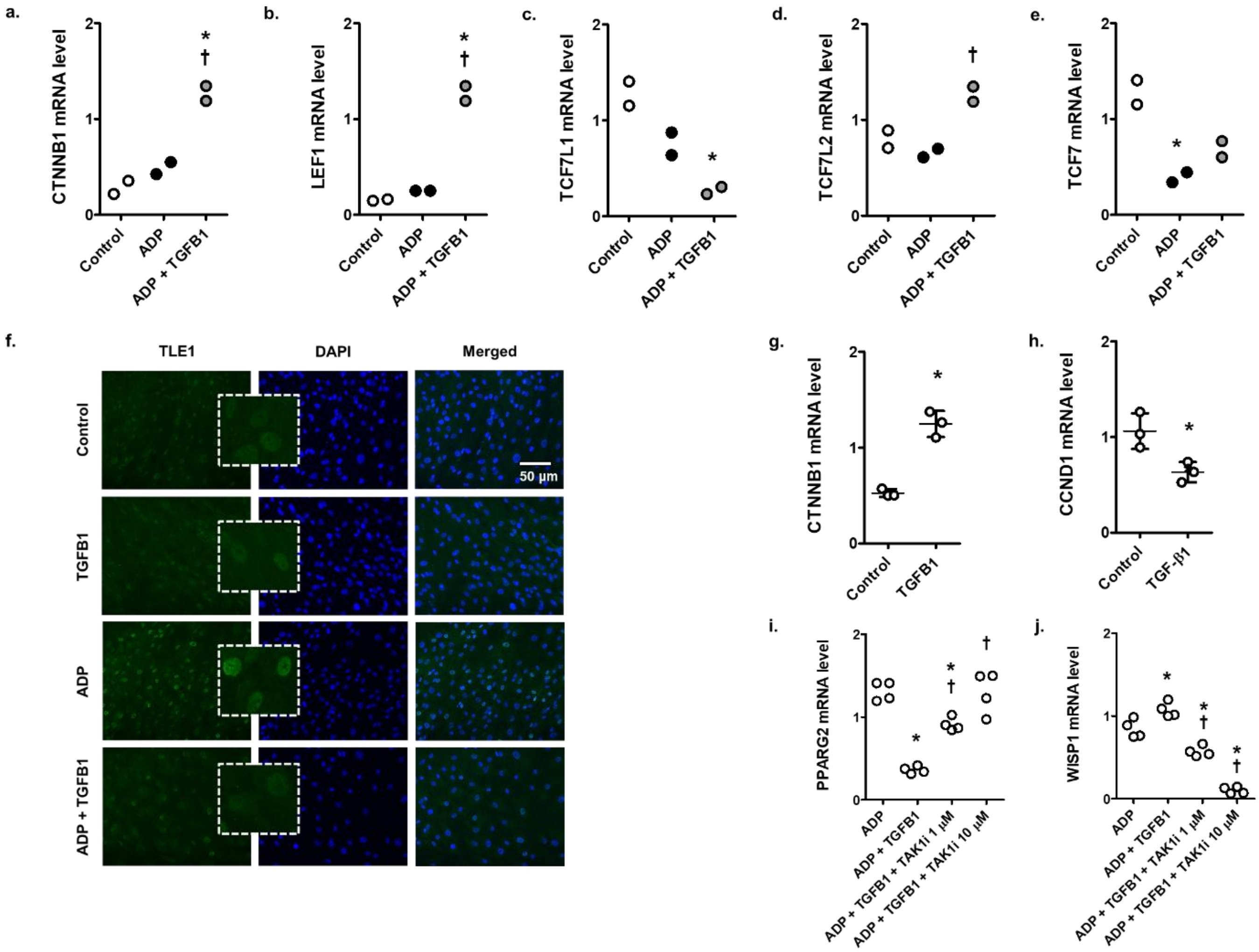

Supplementary Figure S21: ***TGFB1 modulates canonical Wnt regulators.***

(a-e) Bone marrow-derived progenitor cells (bm-MPCs) were cultured in adipogenic media (ADP) with or without TGFB1 (10 ng/mL) for 72 hours. mRNA levels of canonical Wnt pathway in bm-MPCs were measured [Data normalized to *ACTB*, *B2M*, *GAPDH*, *HPRT1*, and *RPLP0*; n = 2; each data point represents an independent sample; ANOVA followed by Bonferroni post hoc analysis: * p<0.05 compared with control, † p<0.05 compared with ADP]. (f) Immunofluorescence staining of bm-MPCs for TLE1 (green). Cells were cultured in ADP media, TGFB1 (10 ng/mL), or ADP media containing TGFB1 (10 ng/mL) for 72 hours. Cells were counterstained with DAPI (blue) [scale bar = 50 µm]. (g, h) mRNA levels of Wnt signaling response genes in bm-MPCs cultured in TGFB1 (10 ng/mL) for 72 hours [Data normalized to *ACTB*; Mean ± SD; n = 3; each data point represents an independent sample; two-tailed student's t-test: * p<0.05 compared with control]. (i, j) bm-MPCs were cultured in adipogenic media (ADP) with TGFB1 (10 ng/mL) and (5Z)-7-Oxozeaenol (TAK1 inhibitor; TAK1i; 1 and 10 µmol/L) for 72 hours. mRNA levels of *PPARG2* (i) and *WISP1* (j) were determined [Data normalized to *ACTB*; n = 4; each data point represents an independent sample; ANOVA

followed by Bonferroni post hoc analysis: * $p<0.05$ compared with ADP, † $p<0.05$ compared with ADP+TGFB1].

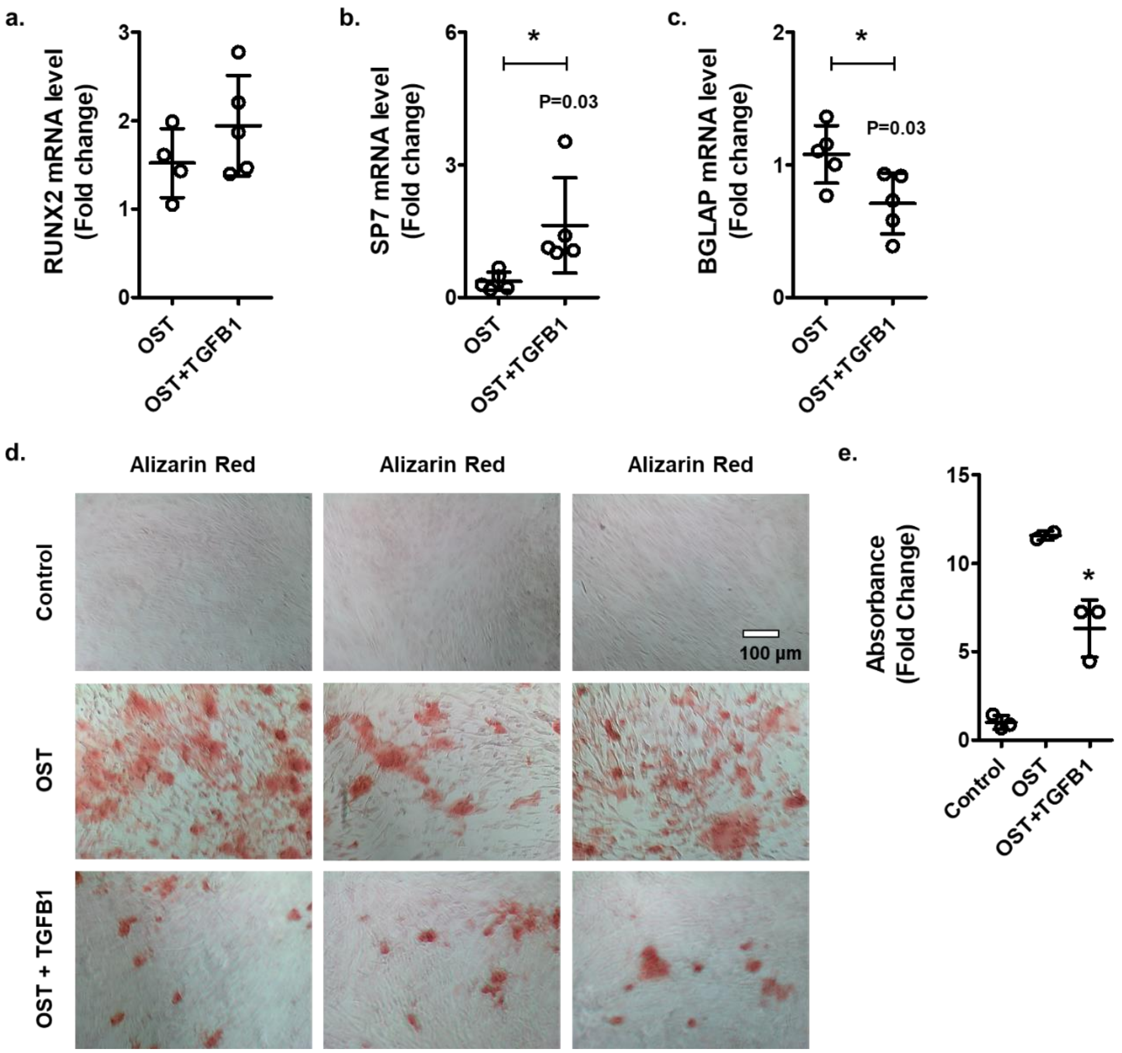

Supplementary Figure S22: ***TGFB1 inhibits late osteogenic differentiation factor in bm-MPCs and may hinder mineralization.***

Bone marrow-derived mesenchymal progenitor cells (bm-MPCs) were cultured in osteogenic media (OST) with or without TGFB1 (10 ng/mL) for 9 days. (a-c) mRNA levels of osteogenesis-associated transcription factors [Data normalized to *ACTB*, *GAPDH*, and *RPLP0;* Mean ± SD; n = 4 OST and 5 OST+TGFB1 in panels a and b, 5 OST and 5 OST+TGFB1 in panel c; each data point represents an independent sample; two-tailed student's t-test: * p<0.05]. (d) bm-MPCs cultured in osteogenic media (OST) with TGFB1 (10 ng/mL) for 9 days were assessed for mineralized matrix and calcium deposition by Alizarin Red S staining [representative of n=3; n is an independent sample; scale bar = 100 µm]. (e) Quantification of the Alizarin Red staining S was performed by measuring absorbance at 405 nm [Data normalized to control conditions; Mean ± SD; n = 3; each data point represents an independent sample; two-tailed student's t-test: * p<0.05 compared with OST].

Supplemental References

Supplemental references relate to primer sequences for qPCR (Supplementary Table S7).

1. Zhong, L., *et al.* Single cell transcriptomics identifies a unique adipose lineage cell population that regulates bone marrow environment. *Elife* **9**(2020).
2. Khimani, A.H., *et al.* Housekeeping genes in cancer: normalization of array data. *Biotechniques* **38**, 739-745 (2005).
3. Lee, P.D., Sladek, R., Greenwood, C.M. & Hudson, T.J. Control genes and variability: absence of ubiquitous reference transcripts in diverse mammalian expression studies. *Genome Res* **12**, 292-297 (2002).
