# Supplementary material for: Dysregulated transforming growth factor-beta mediates early bone marrow dysfunction in diabetes
Source: Commun Biol. 2022 Oct 28;5:1145. doi: 10.1038/s42003-022-04112-2 (PMC9616825; doi:10.1038/s42003-022-04112-2)
Supplement: Supplementary file 3 — Description of Additional Supplementary Data [file 42003_2022_4112_MOESM3_ESM.docx]

**Description of Additional Supplementary Files**

**File name:** Supplementary Data 1

**Description:** The source data behind the main graphs in the paper.
