# Supplementary material for: Dysregulated transforming growth factor-beta mediates early bone marrow dysfunction in diabetes
Source: Commun Biol. 2022 Oct 28;5:1145. doi: 10.1038/s42003-022-04112-2 (PMC9616825; doi:10.1038/s42003-022-04112-2)
Supplement: Supplementary file 5 — Reporting Summary [file 42003_2022_4112_MOESM5_ESM.pdf]

nature portfolio

Corresponding author(s): Zia A. KHAN
Christopher J. HOWLETT

Last updated by author(s): Oct 1, 2022

Reporting Summary

Nature Portfolio wishes to improve the reproducibility of the work that we publish. This form provides structure for consistency and transparency in reporting. For further information on Nature Portfolio policies, see our Editorial Policies and the Editorial Policy Checklist.

Statistics

For all statistical analyses, confirm that the following items are present in the figure legend, table legend, main text, or Methods section.

n/a	Confirmed	
☐	☒	The exact sample size (*n*) for each experimental group/condition, given as a discrete number and unit of measurement
☐	☒	A statement on whether measurements were taken from distinct samples or whether the same sample was measured repeatedly
☐	☒	The statistical test(s) used AND whether they are one- or two-sided<br>*Only common tests should be described solely by name; describe more complex techniques in the Methods section.*
☒	☐	A description of all covariates tested
☐	☒	A description of any assumptions or corrections, such as tests of normality and adjustment for multiple comparisons
☐	☒	A full description of the statistical parameters including central tendency (e.g. means) or other basic estimates (e.g. regression coefficient) AND variation (e.g. standard deviation) or associated estimates of uncertainty (e.g. confidence intervals)
☒	☐	For null hypothesis testing, the test statistic (e.g. *F*, *t*, *r*) with confidence intervals, effect sizes, degrees of freedom and *P* value noted<br>*Give P values as exact values whenever suitable.*
☒	☐	For Bayesian analysis, information on the choice of priors and Markov chain Monte Carlo settings
☒	☐	For hierarchical and complex designs, identification of the appropriate level for tests and full reporting of outcomes
☒	☐	Estimates of effect sizes (e.g. Cohen's *d*, Pearson's *r*), indicating how they were calculated

Our web collection on statistics for biologists contains articles on many of the points above.

Software and code

Policy information about availability of computer code

Data collection	No custom algorithms or software was utilized to obtain data. For transcriptome analysis of cultured bone marrow-derived cells, we used Database for Annotation, Visualization and Integrated Discovery (DAVID) online bioinformatics program (URL is provided in the Methods section. For mouse tissue staining measurements, we used Picro Sirius Red ImageJ macro (URL provided in Methods section). For bone morphometric analyses, we used MarrowQuant (QuPath plugin) and QuPath (freely available, identified in Methods section).
Data analysis	Platforms identified in Data Collection were used for analysis. All URLs are included in the Methods section.

For manuscripts utilizing custom algorithms or software that are central to the research but not yet described in published literature, software must be made available to editors and reviewers. We strongly encourage code deposition in a community repository (e.g. GitHub). See the Nature Portfolio guidelines for submitting code & software for further information.

Data

Policy information about availability of data

All manuscripts must include a data availability statement. This statement should provide the following information, where applicable:

- Accession codes, unique identifiers, or web links for publicly available datasets
- A description of any restrictions on data availability
- For clinical datasets or third party data, please ensure that the statement adheres to our policy

All data figures show raw/individual data points. Source data for the main figures is provided. Gene profiling data is deposited in public repository and available to

researchers (GSE184612). Data is accessible from: https://www.ncbi.nlm.nih.gov/geo/query/acc.cgi?acc=GSE184612.

Reagents and other research material used in this study, if not depleted, will be provided to other researchers upon request.

Human research participants

Policy information about studies involving human research participants and Sex and Gender in Research.

Reporting on sex and gender	N/A
Population characteristics	N/A
Recruitment	N/A
Ethics oversight	N/A

Note that full information on the approval of the study protocol must also be provided in the manuscript.

Field-specific reporting

Please select the one below that is the best fit for your research. If you are not sure, read the appropriate sections before making your selection.

☒ Life sciences ☐ Behavioural & social sciences ☐ Ecological, evolutionary & environmental sciences

For a reference copy of the document with all sections, see nature.com/documents/nr-reporting-summary-flat.pdf

Life sciences study design

All studies must disclose on these points even when the disclosure is negative.

Sample size	An a priori analysis was not performed. Studies outlined in the present study build on previously documented changes in the same diabetes mouse model and hyperglycemia-mimicking conditions in cultured cells. For in vitro studies, we used sample size estimation from our previous studies utilizing the same cell types and protocols, including modulation of adipogenic differentiation (PMID: 22701703, 24496952). These published showed statistically significant changes in gene expression upon modulation of adipogenic differentiation. Same platform was used in the current study. In addition to the analysis of at least 3 experimental replicates, we performed our studies in at least 4 biological replicates (donors). For in vivo studies, published studies have shown statistically different gene expression (n = 3-4, PMID 16249509) and cell composition changes (n = 5; PMID: 20655908) in the retina. Similarly, renal alterations, due to diabetes, are detectable with n = 6-8 (PMID: 17456853). All citations are provided. Based on these studies, we selected the same sizes needed to document changes in our treatment groups.
Data exclusions	No data was excluded.
Replication	All tissues were analyzed from mice (no exclusions). For cell-based studies, all studies were repeated in at least 4 biological replicates (ie donors; details, including lot numbers, are provided in the Methods section. Small 'n' represents an independent sample, not a technical replicate or an additional reading of the same sample.
Randomization	Mice were divided into two groups, diabetic and non-diabetic, by ensuring the body weights were similar between the two groups. Each mouse received a unique identifier (cage#, mouse#). Each cage contained a mixture of diabetic and non-diabetic mice.
Blinding	Mice were assigned a unique identifier (cage, mouse number). All samples (bones and other tissues) were obtained and only unique identifier was transferred to tubes and cassettes containing the tissue. Tissues were stained and analyzed. When plotting the figures, unique identifiers were consulted to accurately display data as diabetic and non-diabetic experimental groups.

Reporting for specific materials, systems and methods

We require information from authors about some types of materials, experimental systems and methods used in many studies. Here, indicate whether each material, system or method listed is relevant to your study. If you are not sure if a list item applies to your research, read the appropriate section before selecting a response.

Materials & experimental systems

n/a	Involved in the study	
☐	☒	Antibodies
☒	☐	Eukaryotic cell lines
☒	☐	Palaeontology and archaeology
☐	☒	Animals and other organisms
☒	☐	Clinical data
☒	☐	Dual use research of concern

Methods

n/a	Involved in the study	
☒	☐	ChIP-seq
☒	☐	Flow cytometry
☒	☐	MRI-based neuroimaging

Antibodies

Antibodies used	All antibodies (and figures resulting from the use of the antibodies) are presented in Supplementary Table S3. The data includes commercial source/vendor, catalogue number, and tissue processing steps.
Validation	Antibodies were first selected based on documented evidence of reactivity in published studies (reactivity to mouse and in immunohistochemistry applications). Following procurement, antibodies were tested in mouse tissues (heart, kidney, bone marrow, retina) and human embryonal carcinoma (for stem cell antigens). Antigen retrieval was tested with Citrate (pH 6) and Tris-based (pH 9) buffers, and temperatures ranging from 60-120 degree C. Negative control was included and entailed omitting primary antibody.

Animals and other research organisms

Policy information about studies involving animals; ARRIVE guidelines recommended for reporting animal research, and Sex and Gender in Research

Laboratory animals	Four-week-old male C57BL/6 mice were used.
Wild animals	N/A
Reporting on sex	Only male mice were used in the study, and clearly indicated in the Methods section.
Field-collected samples	N/A
Ethics oversight	Animal studies were initiated after receiving approval from the University of Western Ontario Animal Care and Veterinary Services (London, Ontario, Canada). Animal Use Protocol: #2019-125.

Note that full information on the approval of the study protocol must also be provided in the manuscript.
